# PROCURE European consensus on breast cancer multigene signatures in early breast cancer management

**DOI:** 10.1038/s41523-023-00510-9

**Published:** 2023-02-24

**Authors:** Giuseppe Curigliano, Fatima Cardoso, Michael Gnant, Nadia Harbeck, Judy King, Anne-Vibeke Laenkholm, Frédérique Penault-Llorca, Aleix Prat

**Affiliations:** 1 European Institute of Oncology, IRCCS, Milan, Italy; 2 Department of Oncology and Hemato-Oncology, University of Milano, Milan, Italy; 3 Breast Unit, Champalimaud Clinical Centre/Champalimaud Foundation, Lisbon, Portugal; 4 Comprehensive Cancer Center, Medical University of Vienna, Vienna, Austria; 5 Breast Center, LMU University Hospital, Munich, Germany; 6 Royal Free Hospital NHS Foundation Trust, London, UK; 7 Zealand University Hospital, Roskilde, Denmark; 8 Centre de Lutte Contre le Cancer Jean Perrin, Imagerie Moléculaire et Stratégies Théranostiques, Université Clermont Auvergne, UMR 1240 INSERM-UCA, Clermont Ferrand, France; 9 Hospital Clínic de Barcelona, August Pi i Sunyer Biomedical Research Institute (IDIBAPS), University of Barcelona, Barcelona, Spain

**Keywords:** Breast cancer, Breast cancer, Prognostic markers, Gene expression, Predictive markers

## Abstract

Breast cancer multigene signatures (BCMS) have changed how patients with early-stage breast cancer (eBC) are managed, as they provide prognostic information and can be used to select patients who may avoid adjuvant chemotherapy. Clinical guidelines make recommendations on the use of BCMS; however, little is known on the current use of BCMS in clinical practice. We conduct a two-round Delphi survey to enquire about current use and perceived utility for specific patient profiles, and unmet needs of BCMS. Overall, 133 panellists experienced in breast cancer across 11 European countries have participated, most using BCMS either routinely (66.2%) or in selected cases (27.1%). Our results show that BCMS are mainly used to assess the risk of recurrence and to select patients for adjuvant chemotherapy; notably, no consensus has been reached on the lack of utility of BCMS for selecting the type of chemotherapy to administer. Also, there are discrepancies between the recommended and current use of BCMS in clinical practice, with use in certain patient profiles for which there is no supporting evidence. Our study suggests that physician education initiatives are needed to ensure the correct use and interpretation of BCMS to, ultimately, improve management of patients with eBC.

## Introduction

Breast cancer is the most common cancer in women, with an estimated 2.3 million new cases diagnosed, 0.68 million deaths, and a 5-year prevalence of 7.8 million women worldwide in 2020^1^. Breast cancer mortality has declined over the past few decades in the majority of European countries^2^ and in North America^3^, due to multidisciplinary and specialised care, better screening modalities for early diagnosis, and major advances in systemic therapy^2,4,5^.

Treatment decision-making in breast cancer previously relied on conventional prognostic factors such as lymph node involvement, tumour size and grade, and status of hormone receptors (HR) and human epidermal growth factor receptor 2 (HER2); however, gene expression signatures contributed to more personalised treatment decisions^6^. Breast cancer multigene signatures (BCMS) provide information on survival, risk of recurrence, and treatment benefit from chemotherapy or hormone therapy beyond estimates from stage and conventional pathologic assessment^7^. Recommendations on the use of 5 commercially available BCMS for early-stage breast cancer (eBC) are included in clinical practice guidelines, such as those from the American Society of Clinical Oncology (ASCO)^8^, the National Comprehensive Cancer Network (NCCN)^9^, and the European Society for Medical Oncology (ESMO)^10^. BCMS may be used to evaluate risk of recurrence, guide adjuvant chemotherapy decision-making, or predict benefit of extended adjuvant endocrine therapy (ET); menopausal status and lymph node involvement are taken into account by some guidelines when making recommendations (Supplementary Table 1).

Breast cancer is the cancer type for which gene expression profiles have been most successful^7^. The introduction of BCMS has considerably changed breast cancer clinical practice, enabling fewer patients to receive chemotherapy when a benefit is not likely and more accurately identifying those for whom a benefit is likely greater. BCMS, consequently, can improve physicians’ confidence in their treatment recommendations^1^, reduce unnecessary chemotherapy-related toxicity^6,11^, and relieve patient anxiety^6^; BCMS have also been found to be cost-effective^11–14^.

Despite the change in clinical practice as a result of BCMS findings^6^, there is no detailed account of their current use nor of their perceived utility by physicians. To bridge this knowledge gap, the PROCURE study aims to assess the use of BCMS among experts in breast cancer care in Europe and to seek consensus on the utility of BCMS for treatment decision-making for different profiles of patients with breast cancer, as well as to gather insights on the possible unmet needs that should be addressed.

## Results

### Panellists’ profile and current use of BCMS

Of the 163 European experts who were invited to participate in the Delphi survey, 140 (85.9%) completed the first-round questionnaire, and 133 (81.6%) completed the second round. Table 1 presents the demographic characteristics of the panellists that participated in both rounds. The mean age was 48.8 years; most panellists worked at a teaching hospital (86.5%), were specialised in medical oncology (72.2%), and had more than 10 years of experience in breast cancer (75.9%) (Table 1). Approximately half of the panellists (45.9%) only treated or analysed biopsies from patients with breast cancer (Table 1).

**Table 1:** Panellists’ demographics.

Characteristics	*n* (%) (*N* = 133)
Type of practice (based on teaching)	
Teaching hospital	115 (86.5)
Non-teaching hospital	16 (12.0)
Individual practice	3 (2.3)
Other	2 (1.5)
Type of practice (based on funding source)	
Public	88 (66.2)
Private	16 (12.0)
Public and private	28 (21.1)
Other	1 (0.8)
Position	
Head of department	46 (34.6)
Consultant	83 (62.4)
Other	4 (3.0)
Age, years (%)	
<35	7 (5.3)
35–44	38 (28.6)
45–54	54 (40.6)
≥55	34 (25.6)
Region of practice	
Iberia	37 (27.8)
France	29 (21.8)
Italy	22 (16.5)
United Kingdom	25 (18.8)
DACH	9 (6.8)
Nordic countries	11 (8.3)
Clinical specialty	
Medical oncology	96 (72.2)
Surgical oncology	10 (7.5)
Pathology	16 (12.0)
Gynaecology	7 (5.3)
Other	4 (3.0)
Experience in breast cancer, years	
<5	4 (3.0)
5–10	28 (21.1)
11–15	34 (25.6)
≥15	67 (50.4)
Treats/sees/analyses other cancer patients/biopsies in addition to breast cancer	
Yes	61 (45.9)
No	72 (54.1)

Iberia: Portugal, Spain. Nordic countries: Denmark, Norway, Sweden. DACH: Germany, Austria, Switzerland.

Percentages may not total 100 due to rounding.

Medical oncologists, surgeons, gynaecologists, and others reported seeing, treating, or diagnosing an average of 36 patients with breast cancer weekly, 63.5% of them diagnosed with eBC. Surveyed pathologists reported analysing an average of 26 breast cancer biopsies per week, 53.9% of them from patients with eBC.

BCMS were available in 94.0% of the hospitals where panellists practiced. A majority (66.2%) of panellists reported routinely using BCMS; 73.4% had experience for over 5 years using BCMS. Of the remainder, 27.1% used BCMS only in selected cases, and 0.7% when requested by a colleague. The panellists who practiced in hospitals without BCMS (6.0%) reported they would use them if available. Notably, 14.3% of panellists reported using BCMS more frequently during the COVID-19 pandemic, especially for patients with lymph node involvement and in the neoadjuvant setting on core biopsies.

### Current clinical practice in eBC and use of BCMS

The factors that panellists regarded as most important when making decisions on adjuvant treatment were international guidelines (40.6% of panellists), national guidelines (39.9%), and their institutional multidisciplinary tumour board (38.4%); other options were hospital guidelines, their own experience, their colleagues’ advice, or other. The use of BCMS was defined by hospital/country guidelines for 85.0% of panellists. The main criteria for using BCMS differed between hospitals with or without guidelines for this purpose (Table 2).

**Table 2:** Main criteria reported by panellists to inform the use of BCMS in hospitals with and without BCMS guidelines.

	Hospitals with BCMS guidelines (*n* = 113)	Hospitals without BCMS guidelines (*n* = 20)
Lymph node involvement	92.0%	80.0%
HER2- by ICH/FISH/CISH	87.6%	70.0%
Tumour size	77.9%	50.0%
Tumour grade	74.3%	55.0%
Spectrum of % ER expression	64.6%	60.0%
Spectrum of % PR expression	50.4%	55.0%
Uncertainty about chemotherapy benefit	55.8%	60.0%
Menopausal status	46.0%	70.0%
% Ki67 expression	62.0%	70.0%
Patient age	44.3%	55.0%
Luminal B surrogate breast cancer	31.0%	35.0%
Uncertainty about ET benefit	8.9%	15.0%
Clinical-pathological algorithms (e.g., Adjuvant Online, NPI)	31.0%	15.0%
Vascular infiltration of tumour	9.7%	5.0%
Other	2.7%	0.0%

*BCMS* breast cancer multigene signatures, *CISH* chromogenic in situ hybridisation, *ER* oestrogen receptor, *ET* endocrine therapy, *HER2* human epidermal growth factor receptor 2, *IHC* immunohistochemistry, *FISH* fluorescent in situ hybridisation, *NPI* Nottingham Prognostic Index, *PR* progesterone receptor.

**Fig. 1:**
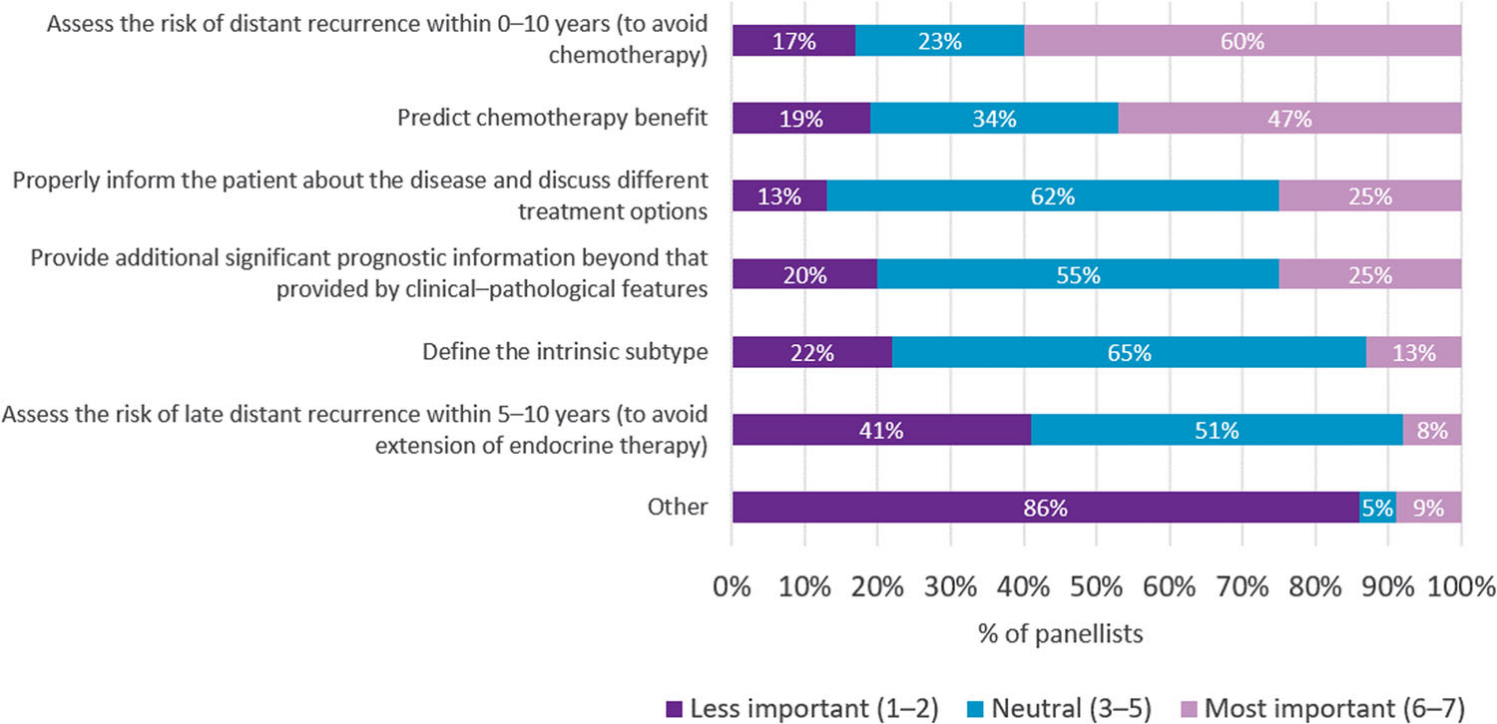
Reasons for use of breast cancer multigene signatures in patients with early-stage breast cancer. Percentage of panellists ranking the importance of reasons for using BCMS from least important (1) to most important (7).

The two main reasons the panellists considered for using BCMS in patients with eBC were to assess the risk of distant recurrence within 10 years in order to avoid chemotherapy (60.2%) and or to predict the benefit from chemotherapy (46.6%) (Fig. 1). Assessment of the risk of late distant recurrence within 5–10 years was considered of less importance by 40.7% of panellists.

**Fig. 2:**
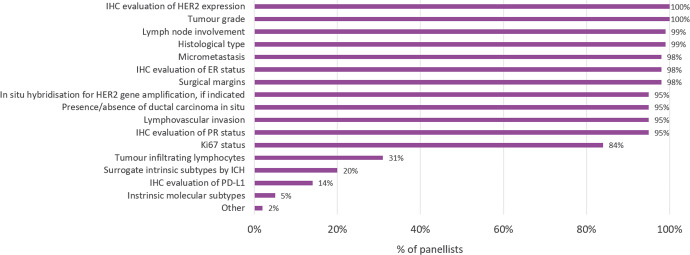
Information included in the pathology or surgical sample report of patients with breast cancer. Percentage of panellists stating the parameters that are included in the report. ER oestrogen receptor, IHC immunohistochemistry, PR progesterone receptor.

When asked about the information contained in the pathology or surgical sample report, panellists noted that common histopathologic features (e.g., tumour grade, lymph node involvement, histologic type) were routinely included in the report (Fig. 2). Hormone receptor status by immunohistochemistry (IHC) was also routinely obtained for oestrogen receptor (ER) (98.5%) and progesterone receptor (PR) (94.7%). HER2 status was evaluated by IHC (100%), with in situ hybridisation (ISH) if indicated (95.5%). Notably, 84.2% of panellists reported Ki67 status to be included in the report, but only 20.0% mentioned surrogate intrinsic subtypes by IHC.

**Fig. 3:**
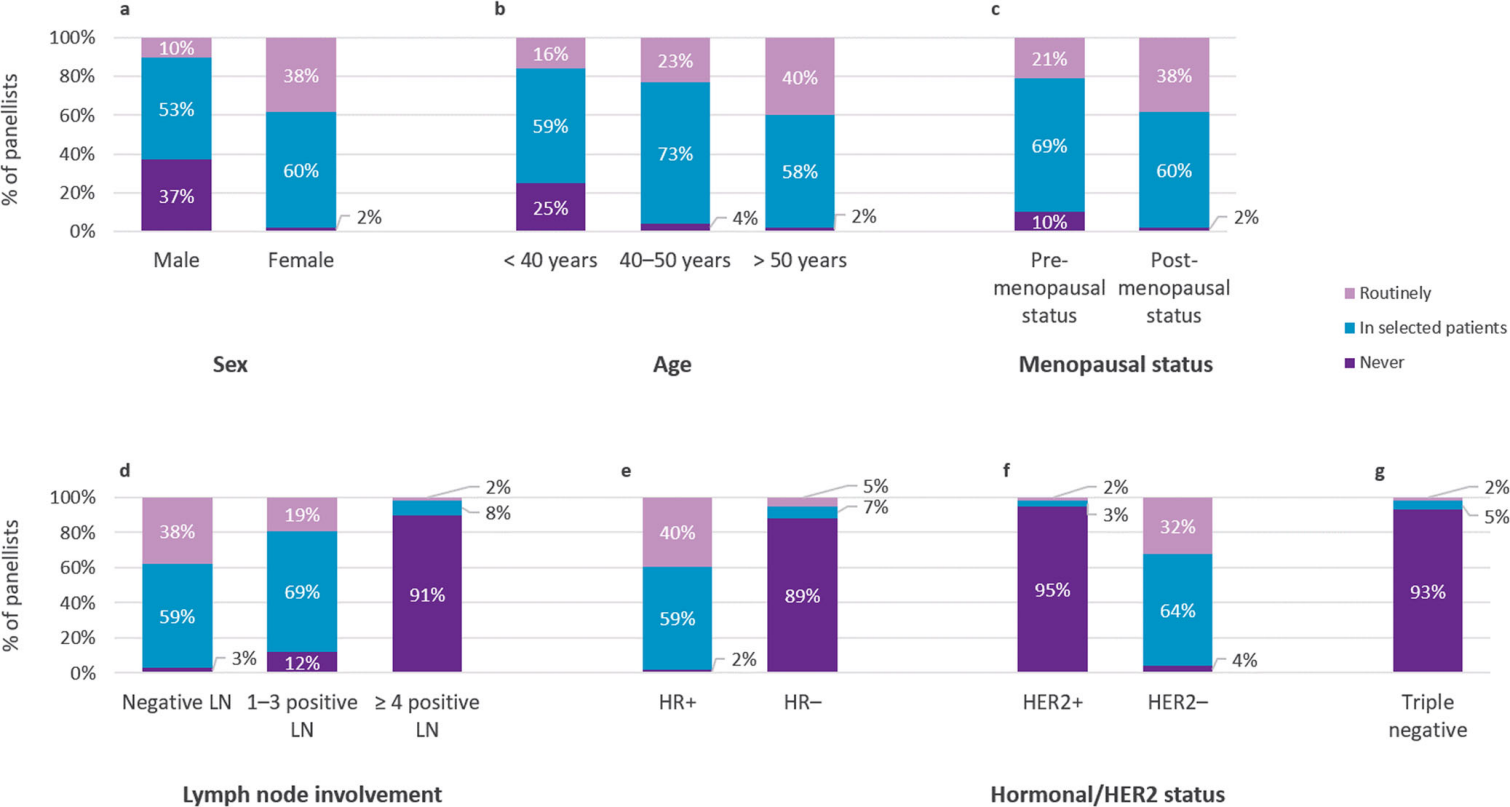
Use of breast cancer multigene signatures to define prognosis and treatment decisions. Percentage of panellists stating the patient profiles with early breast cancer for which they use BCMS to define prognosis and treatment needs. LN lymph node, PR progesterone receptor.

Reported use of BCMS to define prognosis and treatment needs was high (over 88% of panellists, routinely or selectively) in the following profiles of patients with eBC (Fig. 3): women (98.5% of panellists); age over 40 years (96.2% for ages 40–50; 97.7% for age >50), regardless of menopausal status; lack of lymph node involvement (97.0%) or involvement of 1–3 lymph nodes (88.0%); HR + status (98.5%); and HER2- status (96.2%). Some panellists reported using BCMS for patients diagnosed with local recurrence (19.6%) or in the neoadjuvant setting (18.1%), the latter mostly to avoid chemotherapy (83.33%) and to define the intrinsic molecular subtype (79.17%). Also, 25.6% of panellists reported using BCMS in situations that, to their knowledge, were not recommended by clinical guidelines, citing cases such as local recurrence, lymph node involvement, neoadjuvant setting, and HER2 + status. On another note, 70.7% of panellists indicated that they classified patients with micrometastasis as N0.

### Utility of BCMS in eBC based on patient profiles

After the second Delphi round, consensus on the utility of BCMS in profiles of patients with eBC was gained in an additional 4 statements (S1, S14, S33, and S35) and lost in 2 (S15, S16) compared with the first round, resulting in an overall consensus on 16 of the 35 statements in this section (Supplementary Table 2).

Panellists agreed on the utility of tumour intrinsic molecular subtypes determined by gene expression profiling (S1), but not on surrogate intrinsic subtypes determined by IHC (S2). There was consensus on the clinical utility that breast cancer intrinsic molecular subtypes provide for assessing prognosis or residual risk of recurrence with standard of care in HR-positive eBC (S3) and for identifying patients who can safely avoid chemotherapy (S4).

When assessing the risk of recurrence in eBC, panellists agreed that the following aspects of BCMS were very important: evidence from prospective randomised trials (S8), inclusion in guidelines (S9), provision of accurate genomic scores (to also make decisions on use of chemotherapy) (S10), use of formalin-fixed paraffin-embedded tissue samples (S11), consideration of clinical parameters along with gene expression (S12), quick results (S13), and cost-effectiveness (S14). No consensus was reached on regulatory endorsement of BCMS (S15, S16), among others (S17–S22).

The prognostic value of BCMS was considered to be very important for making decisions on the use of chemotherapy in the adjuvant setting—both in patients with no lymph node involvement (S23) and in those with 1–3 positive lymph nodes (S25)—to avoid distant recurrence (within 10 years) or late distant recurrence (within 5–10 years). Consensus was not reached on the use of extended ET in eBC, regardless of the degree of lymph node involvement (S24, S26).

BCMS was considered very useful in patients with post-menopausal eBC (S27); it was considered not useful in the metastatic setting (S33), patients with triple-negative eBC (S34), and in patients with eBC and HER2 + disease (S35). Consensus was not reached on the utility of BCMS in patients with pre-menopausal eBC (S28), male patients with eBC (S30), the neoadjuvant setting (S32), eBC before neoadjuvant treatment (S31), or other histological eBC subtypes beyond invasive ductal carcinoma (S29).

### Use of BCMS in clinical practice

After the second Delphi round, an additional 4 statements (S40, S41, S52, and S56) achieved consensus on the use of BCMS in clinical practice, resulting in an overall consensus on 15 of the 23 statements in this section (Supplementary Table 3).

Panellists agreed with most of the general statements regarding BCMS, including the need for physicians to also take account of the pathological features of breast cancer (S36) as well as the need for BCMS to be based on evidence from randomised clinical trials (S37), provide prognostic and predictive information (S38), guide decision-making on the cost-effective use of adjuvant chemotherapy (S39), produce results quickly (S40), and give information on the risk of late distant recurrence (S41). There was no consensus on the value of second-generation BCMS (S42, S43) for eBC or on the need for BCMS to provide information on the intrinsic molecular subtype (S44).

Consensus was not reached on whether BCMS must be used in all patients to plan locoregional or systemic treatment when eBC is diagnosed/suspected (S45), in all patients with ER + /HER2- eBC after surgery to define the risk of recurrence and the most suitable treatment (S46), or for repeating a BCMS in all patients with ER + /HER2- eBC when locoregional recurrence occurs (S47). The panellists reached consensus disagreement with recommending BCMS testing if breast cancer was only suspected (S48).

Panellists considered that patients have a right to access BCMS results (S49). There was consensus on the need for oncologists (S50), pathologists (S51), and patients (S52)—but not nurses (S54)—to receive training/education on BCMS. Hospitals were also considered by panellists to need to define policies on use of BCMS based on guidelines (S53).

Concerning the classification of tumour subtypes, IHC was considered imperfect on how it defines luminal A and B subtypes (S55) and insufficient to provide surrogates for intrinsic molecular subtypes (S56). There was consensus on the clinical utility of PAM50 intrinsic molecular subtype classification (S57). No consensus was reached on the clinical relevance of the discordance between IHC-based surrogate intrinsic subtypes and PAM50 intrinsic molecular subtypes (S58).

### Unmet needs and future applications of BCMS

Patient profiles were discussed in terms of the need for validated BCMS to evaluate the risk of distant recurrence, assess prognosis, or predict treatment benefit. Consensus was only reached after the second round, in 4 of the 12 items that were asked (Supplementary Table 4). There was consensus on the need for BCMS to predict treatment benefit in patients with ER + advanced/metastatic breast cancer (S60), patients with triple-negative eBC (S66), and in the neoadjuvant setting (S70), and to evaluate the risk of recurrence in the neoadjuvant setting (S69). However, no consensus was reached on the need for BCMS to give accurate prognosis for patients with ER + advanced and/or metastatic breast cancer (S59), HER2 + advanced breast cancer (S63), or triple-negative advanced breast cancer (S67).

## Discussion

In this study, a group of 133 European experts in breast cancer care from a range of medical specialities reported their current use of BCMS in eBC, their opinion on the utility of BCMS, and the unmet needs in this field, reaching consensus on half of the statements posed (35/70). Use of BCMS has increased over time^15^; however, adoption of BCMS in Europe has been delayed compared with the US^16^, and it is encouraging to find that most panellists widely used BCMS in eBC, either routinely or in selected cases. An important finding, however, is that 6% of panellists did not have access to BCMS at their hospital, which highlights disparities in healthcare access for large numbers of patients in Europe and, given the value of BCMS in treatment decision-making, suggests many patients may be exposed to overuse of chemotherapy and avoidable toxicity.

Treating within a multidisciplinary team improves patient outcomes, including survival^5^, and ESMO guidelines recommend a multidisciplinary tumour board to make decisions on adjuvant treatment^10^; however, only 38.4% of panellists considered this to be one of the three most important factors (out of seven) in this context. About 40% of panellists attached similar importance to national and international clinical guidelines for adjuvant treatment decision-making. For 15.0% of panellists, there were no hospital-mandated policies or guidelines for BCMS use, suggesting that they followed national or international guidelines. Notably, the criteria that guided use of BCMS were different between hospitals with and without guidelines. Lymph node involvement was the most common criterion used by hospitals with no policies or guidelines, in line with current recommendations; however, HER2- status and ER status were considered less important than we anticipated. Despite the International Ki67 in Breast Cancer Working Group attributing limited clinical utility to Ki67 expression for treatment decision-making^17^, it guided BCMS use in over two-thirds of hospitals where panellists practiced. In our opinion, Ki67 should be evaluated at experienced laboratories to ensure reproducibility of results, which may underlie its regarded value. The stance on Ki67 may also be due to clinical guidelines’ acknowledgement of its predictive value in certain cases^10^, as well as to the supportive discussion and consensus on its role at the 2021 St Gallen International Breast Cancer Consensus Conference, where there was controversy on the recommended Ki67 threshold to guide decision-making^18^.

Our findings indicate that clinicians are, overall, aware of the recommended uses of BCMS; nevertheless, we have also identified areas where clinician education is needed to ensure adequate BCMS use. The main reported reasons for use of BCMS (assess risk of recurrence and select patients for adjuvant chemotherapy) are in line with current ESMO recommendations^10^. However, despite BCMS not being able to predict the benefit of specific cytotoxic agents^10^; a surprisingly high number (33.1%) of panellists did not consider BCMS unuseful for this purpose (S7). Moreover, a quarter (25.6%) of panellists reported using BCMS for patient profiles outside of those recommended by guidelines. There is no evidence of, or recommendation for, the use of BCMS for patients with ≥4 positive lymph nodes, triple-negative disease, or HER2 + status; still, some panellists reported using BCMS in these patients, either routinely or in selected cases—a finding we consider a cause for concern. Approximately 20% of panellists reported using BCMS in local recurrence, a situation also not considered in the guidelines, and with scarce supporting data. Notably, fewer panellists reported local recurrence as one of their non-guideline uses of BCMS, suggesting that most of them may not be aware that local recurrence is not guideline-mandated.

Only 8.5% of the panellists used BCMS to assess the risk of late distant recurrence within 5–10 years. Accordingly, there was no consensus on the utility of BCMS for extending ET beyond 5 years (S6) nor on their importance to avoid distant or late distant recurrence with extended ET, irrespective of lymph node involvement (S24, S26). This may be explained by the current debate on the extension of adjuvant ET beyond 5 years^18–21^, and the fact that—despite available clinical trial results^20^—controversy remains regarding the optimal duration of extended ET for individual patients^10^. Modest outcome improvements have been achieved by extending ET, accompanied by an increased risk of osteoporosis and bone fractures; however, certain patients experience greater benefit from prolonged ET, such as those with higher intrinsic risk of recurrence^22^.

BCMS were used by panellists regardless of patient age. In particular, 75.2% of panellists used BCMS in patients under 40. Recent findings from a systematic review show that genomic signatures are useful in decision-making for young patients with breast cancer; however, there is still reluctance in the medical community to use BCMS in these patients^23^. BCMS were developed in cohorts of largely postmenopausal patients, and menopausal status may underlie the decision to use BCMS in certain young patients. On this note, panellists considered BCMS to be very useful in patients with post-menopausal eBC (S27), but no consensus was reached regarding patients with pre-menopausal eBC (S28). The continuing controversy about the true benefit of adjuvant chemotherapy—compared with ET—with ovarian function suppression seen in the latest data of three prospective trials of BCMS (MINDACT, TAILORx and RxPONDER) for premenopausal women^24^ may have contributed to this lack of consensus. This issue may be elucidated with the ongoing OPTIMA study, where premenopausal women with breast cancer receive either chemotherapy followed by ET (tamoxifen or an aromatase inhibitor) or undergo BCMS testing, with those obtaining a low-risk score receiving only ET^25,26^.

Few panellists (18.1%) reported performing BCMS on core biopsies, a surprising finding, given the proven concordance between biopsies and surgical specimens in terms of gene expression profiling with BCMS^27–29^. Another unexpected discovery was the increased use of BCMS during the COVID-19 pandemic reported by 14.3% of panellists, particularly for patients with lymph node involvement. This might be interpreted as an attempt to restrict the use of chemotherapy in-hospital needs during the pandemic, in line with the recommendations of the COVID-19 related guidelines^30^.

The panellists generally acknowledged the superiority and clinical utility of intrinsic molecular subtypes identified with BCMS to classify the tumour biology, evaluate prognosis and make treatment decisions, compared with surrogate subtypes determined by IHC (S1, S2, S55, S56, S57). ESMO guidelines recommend subtyping tumours using IHC, stressing that there is no complete concordance between IHC and molecular profiling in subtype determination^10^. Gene expression profiles may be complementary to IHC and provide additional prognostic and/or predictive information than that obtained with pathology assessment^10,31,32^. In line with this, surrogate intrinsic subtypes determined by IHC were included in the pathology report of only 20.0% of hospitals where panellists practiced. Panellists concurred that intrinsic molecular subtypes were very useful for identifying patients who can safely avoid chemotherapy (S4), as described in guidelines^8,10^, and for assessing prognosis (S3), in line with guideline recommendations^9,10^. There was no consensus on the importance of intrinsic molecular subtypes in assessing risk of recurrence (S17), which suggests more data from prospective trials are needed for help establish their clinical utility. Interestingly, panellists did not reach consensus on the importance of retrospective analysis of prospective randomised studies (S20, S21) when deciding to use BCMS, although level 1 evidence for biomarkers can be achieved either with prospective clinical trials specifically designed to address a biomarker (level 1 A) or with analysis of archived specimens from adequately planned prospective trials (level 1B)^33^. Only two of the five commercially available BCMS (Oncotype DX and MammaPrint) have achieved level 1 A evidence based on prospective randomised clinical trials^10,33–37^. Prospective evaluation of BCMS is currently underway for Prosigna (with the phase 3 OPTIMA study) and EndoPredict (with two prospective observational studies, RESCUE and EXET)^38^.

Finally, regarding future applications of BCMS, findings from this study also highlight areas where future research on BCMS is needed to, among others, elucidate their role in locally recurrent disease, guide their accurate interpretation in premenopausal women, or assess their clinical utility in breast cancer with more than 3 positive lymph nodes.

The primary strengths of this report are the use of a well-established consensus-finding methodology, the inclusion of an international group of panellists experienced in breast cancer with broad expertise in using BCMS, and the high response rate achieved in both rounds of the Delphi process. Although the 163 invited panellists were balanced between the 5 regions considered here, varying response rates skewed the panellist distribution, which may limit the possibility of extrapolating the results to all regions, especially in the DACH region and Nordic countries.

Reimbursement of BCMS can be challenging^16^ and may play a role in the type of BCMS that is selected to evaluate patients. At the time of this study, genomic tests were not reimbursed in Portugal or in some regions in Italy, while only some of the 5 commercially available tests were reimbursed by the health system in France, the UK, and DACH region. In Spain, reimbursement varied between regions: some region reimbursed all five, others only a subset. Hospital policies, guidelines recommendations, and the preferences of the multidisciplinary tumour board may impact these decisions. These differences in healthcare management and reimbursement policies among European countries may have affected panellists’ knowledge and attitudes on BCMS^39^, limiting the accuracy in the responses. In addition, the fact that 86.5% of the participants worked in teaching hospitals could challenge extrapolating the results to the European oncology community.

In conclusion, we believe that there is a need for further evidence that clinicians can rely on. In the context of the dynamic environment of guideline updates, the findings from this study support the need for ongoing education initiatives to ensure that clinicians make adequate use of BCMS and interpret the results correctly to guide treatment decision-making. We hope that these findings increase awareness among clinicians on the importance of following guideline recommendations, the benefits of BCMS, and the patient profiles who do not benefit from them, thereby ensuring an optimal use of resources and adequate treatment decisions. Moreover, the cost-effectiveness of BCMS to guide use of adjuvant chemotherapy in high-risk patients with ER + /HER2- eBC has been demonstrated in several country-specific models^12,13^, further enhancing the clinical and economic value of BCMS for these patients in Europe and North America.

## Methods

### Study design and expert panel

The PROCURE study was conducted in 11 European countries that were grouped into 5 strategic regions: Iberia (Spain and Portugal), France, United Kingdom, DACH (Germany, Austria, and Switzerland), Italy, and Nordic countries (Denmark, Norway, and Sweden).

For the proper development of the project, a scientific committee consisting of 8 experts in breast cancer care and specialised in medical oncology, pathology, or surgery across 8 European countries (Austria, Denmark, France, Germany, Italy, Portugal, Spain, and the United Kingdom) was set up. Members of the scientific committee were selected based on their expertise and their renown within the scientific community to ensure the validity and credibility of the results. Other aspects considered were the number of publications, participation in international and European conferences, and involvement in the development of clinical practice guidelines on breast cancer. At least 1 expert in BCMS from each of the 5 regions considered in the study was included, ensuring region-specific knowledge on use of BCMS and supporting adequate interpretation of the results.

The scientific committee was responsible for defining the criteria to select the Delphi panellists: 1) experience in breast cancer (≥5 years); 2) high volume (>50%) of patients with eBC; 3) at least 1 year of experience with BCMS; and 4) practice in large public hospitals. A balanced sample of 163 European clinicians were invited to participate in the Delphi survey.

### Delphi survey

The Delphi survey was conducted with two consecutive web-based rounds, the first one from December 2020 through February 2021, the second one from April to May 2021. After each round, the results were shared and discussed among the steering committee members by live videoconference.

To develop the Delphi survey a literature search was carried out in databases such as PubMed, MEDLINE and Embase using specific key words (early breast cancer, genetic testing, intrinsic subtypes and Europe). Recent publications, mainly in the last 10 years, were included. The scientific committee reviewed the literature and held a virtual meeting to develop the questions and statements to use in the Delphi survey. Subsequently, the final version of the questionnaire with the work done during the meeting was sent to the committee for review and final approval. This is a systematic and iterative approach to build consensus while maintaining anonymity of responders^40^.

The final questionnaires comprised five sections: 1) panellists’ profile and experience with BCMS; 2) current clinical practice in eBC and use of BCMS; 3) utility of the BCMS in eBC based on patient profiles; 4) recommendations on the use of BCMS in clinical practice; and 5) unmet needs and future applications of BCMS (Supplementary Methods). Sections 1 and 2 consisted of questions with yes/no or multiple-choice answers; sections 3–5 consisted of 70 statements on which consensus was sought.

Only statements on which consensus was not reached in during the first round were asked again during the second round, with no modifications. Given that new evidence on BCMS was published after the first questionnaire, three questions from section 2, focused on current clinical practice, were asked again to assess whether panellists’ responses differed. These questions regarded the existence of hospital/country guidelines, the criteria that inform use of BCMS, and the patient profiles used for BCMS.

The checklist for reporting results of internet e-surveys (CHERRIES) guidelines was followed^41^.

### Data analysis

Descriptive statistics were used to present demographic characteristics and group responses to each statement of the Delphi questionnaires. Statements in sections 3–5 of the Delphi questionnaires followed a Likert-type scale from 1 to 9. Consensus was defined as >70% of participants scoring a statement within one of the following score sub-categories: 1–3 (completely disagree/not important at all/useless), 4–6 (neutral), or 7–9 (completely agree/extremely important/essential). Data was analysed using SPSS v22.

### Reporting summary

Further information on research design is available in the Nature Research Reporting Summary linked to this article.

## Supplementary information

Supplementary Information

Reporting Summary

## Data Availability

The full data set is available from the corresponding author upon request.
