# Supplementary material for: PROCURE European consensus on breast cancer multigene signatures in early breast cancer management
Source: NPJ Breast Cancer. 2023 Feb 24;9:8. doi: 10.1038/s41523-023-00510-9 (PMC9951144; doi:10.1038/s41523-023-00510-9)
Supplement: Supplementary file 1 — Supplementary Information [file 41523_2023_510_MOESM1_ESM.pdf]

Supplementary Table 1. Guideline recommendations for use of breast cancer multigene signatures in hormone receptor-positive/human epidermal growth factor receptor 2-negative disease.

BCMS	ASCO[1]		NCCN[2]		ESMO[3]	
	LN	Evidence quality, strength of recommendation	LN	Category of evidence and consensus	LN	Level of evidence, the strength of recommendation
Oncotype DX	LN-	High, strong	LN-	1	NR	I, A
	LN+ (1–3) and PM	High, strong	LN+ (1–3)	1 (postmenopausal), 2A (premenopausal)	—	—
MammaPrint	LN- or LN+ (1–3) and >50 years	Intermediate, strong	LN- or LN+ (1–3)	1	R	I, A
Prosigna	LN- and PM	Intermediate, moderate	LN- or LN+ (1–3)	2A	R	I, B
EndoPredict	LN- or LN+ (1–3) and PM	Intermediate, moderate	LN- or LN+ (1–3)	2A	R	I, B
Breast Cancer Index	LN- or LN+ (1–3) and 5-year ET without recurrence	Intermediate, moderate	LN-	2A	—	—

ET, endocrine therapy; LN, lymph node; NR, not reported; PM, postmenopausal, R, reported.

Supplementary Table 2. Consensus on the utility of BCMS.

Question	Statement no.	Statement	Consensus decision: Response	% of agreement in 1st round	% of agreement in 2nd round
Regardless of the cost, do you believe that is it useful to know the tumour subtype (intrinsic molecular subtypes by gene expression profiling or surrogate intrinsic subtypes by IHC) for prognosis and treatment decision-making?	S1	Intrinsic molecular subtype by gene expression profiling	Consensus: very useful	66.0	72.2
	S2	Surrogate intrinsic subtype by IHC	No consensus: very useful	58.9	63.9
What is the clinical utility of the information provided by the breast cancer intrinsic molecular subtypes?	S3	Assessing prognosis (or residual risk of recurrence with standard of care) of hormone receptor-positive eBC	Consensus: very useful	75.2	75.9
	S4	Identifying a group of patients that can safely avoid chemotherapy	Consensus: very useful	76.6	75.2
	S5	Recruiting a target population in clinical trials	No consensus: very useful	46.1	52.6
	S6	Avoiding extended administration of endocrine therapy after 5 years	No consensus: not useful	29.1	34.6
	S7	Selecting the most appropriate type of chemotherapy treatment	No consensus: not useful	46.1	66.9
When you are considering performing BCMS to assess the risk of recurrence in eBC, how important are the following characteristics?	S8	The test has evidence from prospective randomised clinical trials	Consensus: very important	87.9	87.2
	S9	The test is included in and recommended by national or international guidelines	Consensus: very important	85.1	84.2
	S10	The test provides accurate genomic scores, which allows risk recurrence assessment and chemotherapy decision-making	Consensus: very important	83.7	83.5
	S11	The test uses FFPE tissue sample	Consensus: very important	72.3	73.7
	S12	The test takes into consideration clinical parameters along with gene expression	Consensus: very important	72.3	72.9
	S13	The test provides quick results (less than 1 week)	Consensus: very important	72.3	72.2

	S14	The test has evidence of its cost-effectiveness (accurate patient classification that allows the reduction of chemotherapy associated costs)	Consensus: very important	69.5	70.7
	S15	The test has regulatory endorsement from CE-IVD	No consensus: very important	70.2	69.2
	S16	The test has regulatory endorsement from the FDA or local authorities	No consensus: very important	70.9	69.2
	S17	The test provides intrinsic molecular subtype classification	No consensus: very important	54.6	66.9
	S18	The test could be performed in the hospital local laboratory	No consensus: very important	56.7	61.7
	S19	The test analyses different groups of genes (e.g., proliferation, apoptosis, invasion, cell cycle, hormone receptor-related)	No consensus: very important	47.5	60.1
	S20	The test relies on retrospective analysis of randomised clinical trials for distant recurrence prognostication	No consensus: very important	42.6	54.9
	S21	The test relies on retrospective analysis of randomised clinical trials for prediction information	No consensus: very important	42.6	50.4
	S22	The test analyses a high number of genes	No consensus: very important	24.8	18.8
**How important is the prognostic value of BCMS when deciding therapeutic choices to avoid distant recurrence (within 10 years) and late distant recurrence (within 5–10 years) in patients with eBC?**	S23	Node-negative disease: Chemotherapy in the adjuvant setting	Consensus: very important	88.7	88.0
	S24	Node-negative disease: Extended endocrine therapy	No consensus: very important	52.5	53.4
	S25	1–3 positive nodes: Chemotherapy in the adjuvant setting	Consensus: very important	75.2	75.2
	S26	1–3 positive nodes: Extended endocrine therapy	No consensus: very important	47.5	52.6
	S27	Patients with post-menopausal eBC	Consensus: very useful	90.1	90.2

**What utility do you allocate to BCMS in the following settings?**	S28	Patients with pre-menopausal eBC	No consensus: very useful	59.6	62.4
	S29	Other histological eBC subtypes beyond invasive ductal carcinoma	No consensus: very useful	39.0	49.6
	S30	Male patients with eBC	No consensus: very useful	34.0	31.6
	S31	In patients with eBC before neoadjuvant treatment	No consensus: very useful	30.5	27.8
	S32	In the neoadjuvant setting	No consensus: not useful	39.0	48.1
	S33	In the metastatic setting	Consensus: not useful	52.5	73.7
	S34	Patients with eBC and triple-negative disease	Consensus: not important	73.0	73.7
	S35	Patients with eBC and a HER2 overexpressed profile	Consensus: not useful	68.8	82.0

No consensus statements had been highlighted in grey.
BCMS, breast cancer multigene signatures; CE-IVD, CE-Marking for In-Vitro Diagnostic Devices Directive; eBC, early-stage breast cancer; FDA, US Food and Drug Administration; FFPE, formalin-fixed paraffin-embedded; HER2, human epidermal growth factor receptor 2; IHC, immunohistochemistry.

Supplementary Table 3. Consensus on the use of BCMS in clinical practice.

Question	Statement no.	Statement	Consensus decision: Response	% of agreement in 1st round	% of agreement in 2nd round
**Please indicate your level of agreement with the following statements related to BMCS recommendations**	S36	In addition to genomic results, physicians must also take into consideration clinical and pathological features of the disease	Consensus: completely agree	90.1	91.0
	S37	BCMS must provide information based on evidence from prospective randomised clinical trials	Consensus: completely agree	85.1	85.0
	S38	BCMS must provide both prognostic and predictive information	Consensus: completely agree	80.9	82.0
	S39	BCMS must provide accurate results to guide cost-effective adjuvant chemotherapy decision-making	Consensus: completely agree	80.9	81.2
	S40	BCMS results must be available quickly (less than 1 week)	Consensus: completely agree	63.1	75.2
	S41	BCMS must provide information on the risk of late distant recurrence within 5–10 years	Consensus: completely agree	66.7	73.7
	S42	Second generation BCMS enhance the prognostic value for distant recurrence and risk stratification in node-positive disease[a]	No consensus: completely agree	60.3	68.4
	S43	Second generation BCMS provide more accurate assessment of the risk of recurrence and prognostic result values[1]	No consensus: completely agree	58.9	66.2
	S44	BCMS must provide the intrinsic molecular subtype information	No consensus: completely agree	51.8	61.7
**Please indicate your level of agreement with the following recommendations in relation to the patient profiles that could benefit from BCMS**	S45	BCMS must be used in all patients to plan locoregional or systemic treatment when eBC is diagnosed/suspected	No consensus: completely disagree	68.8	51.9
	S46	BCMS must be performed in all patients with ER+/HER2- eBC after surgery to define both the risk of recurrence and the most suitable treatment	No consensus: completely disagree	45.4	52.6
	S47	BCMS must be repeated in all patients with ER+/HER2- eBC when locoregional recurrence occurs	No consensus: completely disagree	62.4	68.4

	S48	BCMS must be performed in all patients when breast cancer is suspected	Consensus: Completely disagree	77.3	75.9
**Please indicate your level of agreement with the following general recommendations**	S49	Patients have the right to access to the results of BCMS in order to participate in the treatment decision-making process	Consensus: completely agree	85.1	85.7
	S50	There is a need to train oncologists on BCMS	Consensus: completely agree	83.7	84.2
	S51	There is a need to train pathologists on BCMS	Consensus: completely agree	81.6	82.0
	S52	There is a need to better educate patients on BCMS utility	Consensus: completely agree	62.4	78.9
	S53	Hospitals must define a policy for the use of BCMS according to national and or international guidelines	Consensus: completely agree	76.6	78.2
	S54	There is a need to train nurse specialists on BCMS	No consensus: completely agree	49.6	54.9
**Please indicate your level of agreement with the following statements on the discordance between IHC-based surrogate subtypes and PAM50 intrinsic molecular subtypes**	S55	Current IHC-based definitions of luminal A and B breast cancers are imperfect when compared with multigene expression-based assays	Consensus: completely agree	72.3	73.7
	S56	The intrinsic subtype classification based on IHC is not sufficient to show an adequate surrogate for the genomic subtypes	Consensus: completely agree	64.5	73.7
	S57	The intrinsic molecular subtype classification provided by PAM50 supports its clinical utility	Consensus: completely agree	70.2	70.7
	S58	The discordance between IHC-based surrogate subtypes and PAM50 intrinsic molecular subtypes could explain the under-treatment or over-treatment of patients with breast cancer	No consensus: completely agree	64.5	67.7

No consensus statements had been highlighted in grey.
[1]Oncotype DX and MammaPrint are first-generation BCMS; EndoPredict, Prosigna, and Breast Cancer Index are second-generation BCMS.
BCMS, breast cancer multigene signatures; eBC, early-stage breast cancer; ER, oestrogen receptor; HER2, human epidermal growth factor receptor 2; IHC, immunohistochemistry; PAM50, Prediction Analysis of Microarray 50.

Supplementary Table 4. Consensus on the unmet needs and future applications of BCMS.

Question	Statement no.	Statement	Consensus decision: Response	% of agreement in 1st round	% of agreement in 2nd round
**Please indicate your level of agreement with the need for validated BCMS in the following settings**	S59	ER+ advanced and/or metastatic breast cancer: prognosis	No consensus: completely agree	41.1	50.4
	S60	ER+ advanced and/or metastatic breast cancer: predict treatment benefit	Consensus: completely agree	63.1	78.2
	S61	HER2+ eBC: risk of recurrence	No consensus: completely agree	49.6	57.1
	S62	HER2+ eBC: predict treatment benefit	No consensus: completely agree	55.3	63.9
	S63	HER2+ advanced breast cancer: prognosis	No consensus: completely agree	31.2	21.8
	S64	HER2+ advanced breast cancer: predict treatment benefit	No consensus: completely agree	42.6	51.1
	S65	Triple negative eBC: risk of recurrence	No consensus: completely agree	46.8	63.2
	S66	Triple negative eBC: predict treatment benefit	Consensus: completely agree	50.4	72.2
	S67	Triple negative advanced breast cancer: prognosis	No consensus: completely agree	33.3	36.8
	S68	Triple negative advanced breast cancer: predict treatment benefit	No consensus: completely agree	47.5	59.4
	S69	Neoadjuvant: risk of recurrence	Consensus: completely agree	60.3	71.4
	S70	Neoadjuvant: predict treatment benefit	Consensus: completely agree	67.4	81.2

No consensus statements had been highlighted in grey.
BCMS, breast cancer multigene signatures; eBC, early-stage breast cancer; ER, oestrogen receptor; HER2, human epidermal growth factor receptor 2.

Supplementary Table 5. List of PROCURE Project panellist (by alphabetical order and by country)

COUNTRY	NAME	SURNAME	SPECIALITY
AUSTRIA	SIMON PETER	GAMPENRIEDER	MEDICAL ONCOLOGIST
DENMARK	MARIA	ROSSING	CLINICAL BIOCHEMIST
DENMARK	TRINE	TRAMM	PATHOLOGIST
FRANCE	BARBARA	PISTILLI	MEDICAL ONCOLOGIST
FRANCE	BENOIT	QUILICHINI	PATHOLOGIST
FRANCE	BERNARD	FLIPO	SURGEON
FRANCE	CHRISTELLE	LÉVY	MEDICAL ONCOLOGIST
FRANCE	DELPHINE	GARBAY	MEDICAL ONCOLOGIST
FRANCE	ELISABETH	RUSS	PATHOLOGIST
FRANCE	ELISE	DELUCHE	MEDICAL ONCOLOGIST
FRANCE	EMMANUELLE	CHARAFE	PATHOLOGIST
FRANCE	GILLES	FREYER	MEDICAL ONCOLOGIST
FRANCE	HÉRVE	BONNEFOI	MEDICAL ONCOLOGIST
FRANCE	JEAN CLAUDA	HAMMOU	PATHOLOGIST
FRANCE	JEAN-YVES	PIERGA	MEDICAL ONCOLOGIST
FRANCE	JONATHAN	LOPEZ	MOLECULAR BIOLOGIST
FRANCE	LAURENCE	LANCRY-LECOMTE	MEDICAL ONCOLOGIST
FRANCE	LUCIE	TIXIER DEVES	PATHOLOGIST
FRANCE	MARIANNE	LEHEURTEUR	MEDICAL ONCOLOGIST
FRANCE	MARJORIE	BACIUCHKA	MEDICAL ONCOLOGIST
FRANCE	MONIQUE	COHEN	SURGEON
FRANCE	MONY	UNG	MEDICAL ONCOLOGIST
FRANCE	NAWALE	HAJJAJI	MEDICAL ONCOLOGIST
FRANCE	PAUL	COTTU	MEDICAL ONCOLOGIST
FRANCE	PIERRE	HEUDEL	MEDICAL ONCOLOGIST
FRANCE	PUPPO	SÉVERINE	GYNAECOLOGIST
FRANCE	RÉMY	SALMON	SURGEON
FRANCE	ROMAN	ROUZIER	SURGEON
FRANCE	VINCENT	MASSARD	MEDICAL ONCOLOGIST
FRANCE	WILLIAM	JACOT	MEDICAL ONCOLOGIST
FRANCE	XAVIER	CARCOPINO	SURGEON
FRANCE	YVES MARIE	ROBIN	PATHOLOGIST
GERMANY	ANDREAS	HARTKOPF	GYNAECOLOGIST
GERMANY	HANS	TESCH	MEDICAL ONCOLOGIST
GERMANY	HANS-CHRISTIAN	KOLBERG	GYNAECOLOGIST
GERMANY	MICHAEL PATRICK	LUX	GYNAECOLOGIST
GERMANY	RAMONA	ERBER	PATHOLOGIST
GERMANY	WOLFRAM	MALTER	GYNAECOLOGIST
ITALY	ALBERTO	ZAMBELLI	MEDICAL ONCOLOGIST
ITALY	ANGELO	DI LEO	MEDICAL ONCOLOGIST
ITALY	ANTONIO	FRASSOLDATI	MEDICAL ONCOLOGIST
ITALY	BENEDETTA	CONTE	MEDICAL ONCOLOGIST
ITALY	CARMEN	CRISCITIELLO	MEDICAL ONCOLOGIST
ITALY	CATERINA	MARCHIO	PATHOLOGIST

ITALY	CATIA	ANGIOLINI	MEDICAL ONCOLOGIST
ITALY	DANIELE	GENERALI	MEDICAL ONCOLOGIST
ITALY	FEDERICO	PIACENTINI	MEDICAL ONCOLOGIST
ITALY	FRANCESCO	SCHETTINI	MEDICAL ONCOLOGIST
ITALY	FRANCESCO	DI COSTANZO	MEDICAL ONCOLOGIST
ITALY	GAIA	GRIGUOLO	MEDICAL ONCOLOGIST
ITALY	GIACOMO	PELIZZARI	MEDICAL ONCOLOGIST
ITALY	GIAMPAOLO	BIANCHINI	MEDICAL ONCOLOGIST
ITALY	LAURA	BIGANZOLI	MEDICAL ONCOLOGIST
ITALY	LAURA	CORTESI	MEDICAL ONCOLOGIST
ITALY	MARIA VITTORIA	DIECI	MEDICAL ONCOLOGIST
ITALY	MAURO	MASTROPASQUA	PATHOLOGIST
ITALY	PIERFRANCESCO	FRANCO	RADIATION ONCOLOGIST
ITALY	SERENA	DI COSIMO	MEDICAL ONCOLOGIST
ITALY	VALENTINA	GUARNERI	MEDICAL ONCOLOGIST
ITALY	VINCENZO	ADAMO	MEDICAL ONCOLOGIST
NORWAY	HANS PETTER	EIKESDAL	MEDICAL ONCOLOGIST
NORWAY	HEGE	OMA OHNSTAD	MEDICAL ONCOLOGIST
PORTUGAL	FERNANDO	SCHMITT	PATHOLOGIST
PORTUGAL	ISABEL	PAZOS	MEDICAL ONCOLOGIST
PORTUGAL	JOANA	MOURATO RIBEIRO	MEDICAL ONCOLOGIST
PORTUGAL	NOEMIA	AFONSO	MEDICAL ONCOLOGIST
PORTUGAL	SUSANA	SOUSA	MEDICAL ONCOLOGIST
SPAIN	AMILLANO	KEPA	MEDICAL ONCOLOGIST
SPAIN	ANA	SÁNCHEZ	MEDICAL ONCOLOGIST
SPAIN	ANA	SANTABALLA	MEDICAL ONCOLOGIST
SPAIN	ANA ISABEL	BALLESTEROS	MEDICAL ONCOLOGIST
SPAIN	BÁRBARA	ADAMO	MEDICAL ONCOLOGIST
SPAIN	BEGOÑA	BERMEJO	MEDICAL ONCOLOGIST
SPAIN	CÉSAR	RODRÍGUEZ	MEDICAL ONCOLOGIST
SPAIN	CRISTINA	PÉREZ	MEDICAL ONCOLOGIST
SPAIN	CRISTINA	SAURA	MEDICAL ONCOLOGIST
SPAIN	FEDERICO	ROJO	PATHOLOGIST
SPAIN	GEMMA	VIÑAS	MEDICAL ONCOLOGIST
SPAIN	ISABEL	BLANCAS	MEDICAL ONCOLOGIST
SPAIN	JOSÉ	PONCE	MEDICAL ONCOLOGIST
SPAIN	JOSÉ ENRIQUE	ALÉS	MEDICAL ONCOLOGIST
SPAIN	JOSÉ IGNACIO	CHACÓN	MEDICAL ONCOLOGIST
SPAIN	JOSÉ MANUEL	BAENA	MEDICAL ONCOLOGIST
SPAIN	JUAN	DE LA HABA	MEDICAL ONCOLOGIST
SPAIN	JUAN MIGUEL	CEJALVO	MEDICAL ONCOLOGIST
SPAIN	LOURDES	CALVO	MEDICAL ONCOLOGIST
SPAIN	LUIS	MANSO	MEDICAL ONCOLOGIST
SPAIN	MARÍA	VIDAL	MEDICAL ONCOLOGIST
SPAIN	MIGUEL	GIL	MEDICAL ONCOLOGIST
SPAIN	MIGUEL	MARTÍN	MEDICAL ONCOLOGIST
SPAIN	MIQUEL ÀNGEL	SEGUÍ	MEDICAL ONCOLOGIST
SPAIN	MIREIA	MELÉ	MEDICAL ONCOLOGIST
SPAIN	MIREIA	MARGELÍ	MEDICAL ONCOLOGIST
SPAIN	PEDRO	SÁNCHEZ	MEDICAL ONCOLOGIST

SPAIN	PEDRO LUIS	FERNÁNDEZ	PATHOLOGIST
SPAIN	SARA	LÓPEZ	MEDICAL ONCOLOGIST
SPAIN	SONIA	PERNAS	MEDICAL ONCOLOGIST
SPAIN	TOMÁS	PASCUAL	MEDICAL ONCOLOGIST
SPAIN	XAVIER	GONZÁLEZ	MEDICAL ONCOLOGIST
SWEDEN	ALEXIOS	MATIKAS	MEDICAL ONCOLOGIST
SWEDEN	ANTONIOS	VALACHIS	MEDICAL ONCOLOGIST
SWEDEN	BARBRO KRISTINA	LINDERHOLM	MEDICAL ONCOLOGIST
SWEDEN	CAROLA	ANDERSSON	MOLECULAR BIOLOGIST
SWEDEN	JOHAN	HARTMAN	PATHOLOGIST
SWEDEN	MONIKA	UMINSKA	MEDICAL ONCOLOGIST
SWEDEN	THEODOROS	FOUKAKIS	MEDICAL ONCOLOGIST
SWITZERLAND	CARLOS	VILLENA	SURGEON
SWITZERLAND	RAU	TILMAN	MEDICAL ONCOLOGIST
UNITED KINGDOM	ABEER	SHAABAN	PATHOLOGIST
UNITED KINGDOM	AMNA	SHERI	MEDICAL ONCOLOGIST
UNITED KINGDOM	ANDREAS	MAKRIS	MEDICAL ONCOLOGIST
UNITED KINGDOM	ANNABEL	BORLEY	MEDICAL ONCOLOGIST
UNITED KINGDOM	APOSTOLOS	KONSTANTIS	MEDICAL ONCOLOGIST
UNITED KINGDOM	CAROLINE	OSBORNE	SURGEON
UNITED KINGDOM	CATHERINE	HARPER-WYNNE	MEDICAL ONCOLOGIST
UNITED KINGDOM	CLIONA	KIRWAN	SURGEON
UNITED KINGDOM	DUNCAN	WHEATLEY	MEDICAL ONCOLOGIST
UNITED KINGDOM	ELLEN	COPSON	MEDICAL ONCOLOGIST
UNITED KINGDOM	ELSA	PAPADIMITRAKI	MEDICAL ONCOLOGIST
UNITED KINGDOM	FHARAT	RAJA	MEDICAL ONCOLOGIST
UNITED KINGDOM	GIANFILIPPO	BERTELLI	MEDICAL ONCOLOGIST
UNITED KINGDOM	GIRIJA	ANAND	CLINICAL ONCOLOGIST
UNITED KINGDOM	HENRY	CAIN	SURGEON
UNITED KINGDOM	KELLY	LAMBERT	SURGEON
UNITED KINGDOM	LAI CHENG	YEW	MEDICAL ONCOLOGIST
UNITED KINGDOM	MARK	TUTHILL	MEDICAL ONCOLOGIST
UNITED KINGDOM	MARK	BERESFORD	MEDICAL ONCOLOGIST
UNITED KINGDOM	MAY	TEOH	CLINICAL ONCOLOGIST
UNITED KINGDOM	PANKAJ	ROY	SURGEON
UNITED KINGDOM	ROBERT	LAING	MEDICAL ONCOLOGIST
UNITED KINGDOM	SACHA	HOWELL	MEDICAL ONCOLOGIST
UNITED KINGDOM	STUART	MCINTOSH	SURGEON
UNITED KINGDOM	URMILA	BARTHAKUR	MEDICAL ONCOLOGIST

Supplementary Methods. Delphi Questionnaire (Wave 1 and Wave 2)

PROCURE questionnaire – Wave 1

Thank you very much for your willingness to participate in the PROCURE project.

Before starting, we would like to assure you that your answers will always be confidential, therefore we would appreciate it if you could answer as honestly as possible. The ultimate result is meant to be a true consensus of what the group thinks.

Questions based on opinion will be analysed using the DELPHI methodology, which is an investigational technique frequently used in healthcare research to reach a consensus on different topics among a group of experts. This methodology is based on a questionnaire that is administered twice (1st and 2nd wave). During the 2nd wave, you will see the results from the 1st wave, and those questions where a consensus hasn't been reached in the 1st wave will be asked again.

The questionnaire will be structured into 5 sections:

1. Participant's profile (this section will not be asked again in the 2nd wave).
2. Current daily clinical practice with breast cancer multigene signatures (this section will not be asked again in the 2nd wave).
3. Opinions on the utility of the characteristics of multigene signatures in early breast cancer according to patient profiles.
4. Recommendations on the use of breast cancer multigene signatures in clinical practice.
5. Future applications of breast cancer multigene signatures.

There are no correct nor incorrect answers, please answer according to your current clinical practice and when requested, please express your level of agreement or disagreement on the different statements, based on your expertise and your professional opinion.

Each questionnaire will be available for a maximum period of 5 weeks after launch. The platform allows to save your progress, so you will be able to access the questionnaire as many times as necessary to complete it during this period. Once the end of the period is reached, access to the questionnaire will be closed.

Once again, thank you for your time! The questionnaire will take you about 1h30 to complete. Let's start!

1 Participant profile*

We kindly ask you to reply to the following questions that aim to characterise the participants as well as their expertise in use of breast cancer multigene signatures. All your answers will be kept confidential.

*This section will not be asked again in the 2nd wave.

1.1 Please indicate your medical specialty (single answer)

- ☐ Medical oncologist
- ☐ Surgeon
- ☐ Pathologist
- ☐ Gynaecologist
- ☐ Other, please, specify: ________

1.2 Please, indicate your age

Age: __________

1.3 Do you treat/see/analyse other cancer patients/biopsies in addition to those with breast cancer? (single answer)

- ☐ Yes, I'm also in charge of other types of cancer patients/biopsies.
  - o Please, specify: ___________
- ☐ No. I only treat/see/analyse breast cancer patients/biopsies.

1.4 What kind of centre do you work in? (multiple choice)

- ☐ Teaching hospital
- ☐ Non-teaching hospital
- ☐ Individual office/office with few healthcare professionals
- ☐ Other. Please, specify: ___________

1.5 Do you work at a public, private or both type of centres? (single answer)

- ☐ Public centre
- ☐ Private centre
- ☐ Both
- ☐ Other. Please, specify: ___________

1.6 Which is your current position? (single answer)

- ☐ Head of department
- ☐ Consultant
- ☐ Other. Please, specify: __________

1.7 How long have you been diagnosing/treating/analysing biopsies from patients with breast cancer for? (single answer)

- ☐ < 5 years
- ☐ 5-10 years
- ☐ 11-15 years
- ☐ >15 years

1.8 On average, how many breast cancer patients do you personally see, treat or diagnose on a weekly basis, without taking into consideration the COVID-19 period? (please think about the number of patients, not visits)

_____ Number of patients per week

1.9 What proportion of your breast cancer patients have been diagnosed with early stage breast cancer?
____ % of patients (%)

1.10 On average, how many biopsies do you personally analyse on a weekly basis, without taking into consideration the COVID-19 period?

_____ Number of biopsies per week

1.11 What proportion of the previously mentioned biopsies, are from early stage breast cancer patients?
____ % of biopsies (%)

1.12 Are you familiar with the use of breast cancer multigene signatures? (single answer)

- ☐ I don't have enough experience to answer this question.
- ☐ Yes, I routinely use breast cancer multigene signatures.
- ☐ Yes, I use them only in selected cases.
- ☐ No, my colleagues are in charge of requesting them and the interpretation of the results.
- ☐ No, they are not available where I work.

1.12.1 If these tests were available in your hospital, would you use them?

- ☐ Yes
- ☐ No

1.13 How many years have you been using breast cancer multigene signatures for?
_______ years

1.14 Have you ever used in your routine clinical practice any of the following breast cancer multigene signatures? (multiple choice)

- ☐ Prosigna
- ☐ Oncotype DX
- ☐ MammaPrint
- ☐ EndoPredict
- ☐ Other. Please, specify: _______

2 Current daily clinical practice with breast cancer multigene signatures*

We kindly ask you to answer the following questions with the aim of understand your current clinical practice in breast cancer and the use of breast cancer multigene signatures.

*This section will not be asked again in the 2nd wave.

2.1 How do decide about treatment in the **adjuvant setting**? Please rank them from the least important source (1) to the most (7)

- ☐ My own experience
- ☐ My colleagues' advice
- ☐ International guidelines (ASCO/NCCN/ST. GALLEN/ESMO). Please, specify: _____
- ☐ National guidelines. Please, specify: _____
- ☐ Hospital or country guidelines
- ☐ Multidisciplinary tumour board
- ☐ Other. Please, specify: _____

2.2 Is there a **hospital/country guideline** in your hospital/region that defines when to use a breast cancer multigene signature? (single answer)

- ☐ Yes
- ☐ No

2.3 What are the **criteria defined for the use of breast cancer multigene signatures** in your hospital? (multiple answer)

- ☐ Age of the patient
- ☐ Menopausal status
- ☐ Tumour size
- ☐ Nodal status
- ☐ Spectrum of % of ER expression
- ☐ Spectrum of % of PR expression
- ☐ HER2 negative by IHC/FISH/CISH
- ☐ Clinical-pathological algorithms (Adjuvant Online, NPI, Predict, etc.)
- ☐ Grade of the tumour
- ☐ % of Ki67 expression
- ☐ Vascular infiltration of the tumour
- ☐ Uncertainty about benefit of chemotherapy
- ☐ Luminal B surrogate breast cancer
- ☐ Uncertainty about endocrine therapy benefit
- ☐ Other. Please, specify: ___________

**CISH: chromogenic in-situ hybridisation; ER: Oestrogen receptor; FISH: fluorescent in-situ hybridization; IHC: inmunohistochemistry; PR: progesterone receptor.*

2.4 In the hypothetical situation that you **could choose your preferred breast cancer multigene signature**, which one would you select for your routine clinical practice in most cases? (single answer)

- ☐ Prosigna
- ☐ Oncotype DX
- ☐ MammaPrint
- ☐ EndoPredict
- ☐ It depends on the patient profile
- ☐ Other. Please specify:_________

2.5 What **information does your pathology report or surgical sample report of luminal invasive early breast cancer** routinely contain? (multiple answer)

- ☐ Presence/absence of ductal carcinoma in situ
- ☐ Histological type
- ☐ Tumour grade
- ☐ IHC evaluation of ER status
- ☐ IHC evaluation of the PR status
- ☐ IHC evaluation for HER2 expression
- ☐ In situ hybridisation for HER2 gene amplification, if indicated
- ☐ Ki67 status
- ☐ Surrogate Intrinsic Subtypes
- ☐ Molecular Instrinsic Subtypes
- ☐ Lymph nodes involvement
- ☐ Micrometastasis
- ☐ Surgical margins
- ☐ Lymphovascular invasion

☐ Tumour infiltrating lymphocytes
☐ IHC evaluation of PD-L1
☐ Other. Please, specify: ________________

**ER: Oestrogen receptor; IHC: inmunohistochemistry; PR: progesterone receptor.*

2.6 What are the **main reasons why you use breast cancer multigene signatures** in early breast cancer patients? Please rank them from the least important reason (1) to the most (6)

☐ I do not directly order to perform a breast cancer multigene signature
☐ To assess the risk of distant recurrence in years 0 to 10 (to avoid chemotherapy)
☐ To assess the risk of late distant recurrence in years 5 to 10 (to avoid extension of the endocrine therapy)
☐ To predict chemotherapy benefit
☐ To define the intrinsic subtype
☐ To properly inform the patient about the disease and discuss different treatment options
☐ To provide additional significant prognostic information beyond that provided by clinical-pathological features
☐ Other. Please, specify: _____________

2.7 When do you **use a breast cancer multigene signatures to define the prognosis and treatment needs** in the following ER+/HER2- early breast cancer profiles? (one answer per row)

PATIENT PROFILES	Never	In selected patients	Routinely
**Gender**			
Male	☐	☐	☐
Female	☐	☐	☐
**Age**			
< 40 years old	☐	☐	☐
40 – 50 years old	☐	☐	☐
> 50 years old	☐	☐	☐
**Menopausal status**			
Pre-menopausal status	☐	☐	☐
Post-menopausal status	☐	☐	☐
**Nodal status**			
Negative LN	☐	☐	☐
1 to 3 positive LN	☐	☐	☐
≥ 4 positive LN	☐	☐	☐
**HR status**			
HR positive	☐	☐	☐
HR negative	☐	☐	☐
HER2 status			
HER2 positive	☐	☐	☐
HER2 negative	☐	☐	☐
Triple negative	☐	☐	☐

**HR: hormone receptor; LN: lymph nodes.*

2.8 When do you **use the following breast cancer multigene signatures** in ER+/HER2- early breast cancer patients **to gain additional prognostic and/or predictive information**? (one answer per row. The answer "Others" could remain in blank if needed)

GENE EXPRESSION PROFILES	Not available	Never	In selected patients	Routinely
Prosigna	☐	☐	☐	☐
Oncotype DX	☐	☐	☐	☐
EndoPredict	☐	☐	☐	☐

MammaPrint	☐	☐	☐	☐
Breast Cancer Index	☐	☐	☐	☐
Others. Please, specify:	☐	☐	☐	☐

2.9 Based upon your knowledge and experience' which of the following **characteristics do you attribute to each of the following breast cancer multigene signatures**? (multiple answers per row)

CHARACTERISTICS	Prosigna	Oncotype DX	EndoPredict	MammaPrint	Breast Cancer Index	Other (previously specified)
The test is included in relevant guidelines (e.g., ASCO, ESMO, NCCN)	☐	☐	☐	☐	☐	☐
The test has Level 1 evidence available	☐	☐	☐	☐	☐	☐
The test provides a dichotomous (high/low) result	☐	☐	☐	☐	☐	☐
The test analyses a relevant number of different genes	☐	☐	☐	☐	☐	☐
The test analyses ER response pathway	☐	☐	☐	☐	☐	☐
The test could be performed in the hospital local laboratory	☐	☐	☐	☐	☐	☐
The test provides accurate risk of recurrence to guide chemotherapy decisions	☐	☐	☐	☐	☐	☐
The test takes into consideration clinical parameters along with gene expression	☐	☐	☐	☐	☐	☐
The test has been validated for LN- patients	☐	☐	☐	☐	☐	☐
The test has been validated for LN+ patients	☐	☐	☐	☐	☐	☐
The test relies on prognostic validation trials for late-distant recurrence (in years 5 to 10)	☐	☐	☐	☐	☐	☐
The test evidence includes validation trials for therapy prediction information	☐	☐	☐	☐	☐	☐

The test provides intrinsic subtype classification	☐	☐	☐	☐	☐	☐
The test has cost-effectiveness evidence (accurate patient classification that allows the reduction of chemotherapy associated costs)	☐	☐	☐	☐	☐	☐
The test has regulatory endorsement from the FDA	☐	☐	☐	☐	☐	☐
The test has regulatory endorsement from CE-IVD	☐	☐	☐	☐	☐	☐
The test relies on prognostic validation trials for late-distant recurrence (in years 5 to 10)	☐	☐	☐	☐	☐	☐

**CE-IVD: CE-Marking for In-Vitro Diagnostic Devices Directive; FDA: US Food and Drug Administration; LN: lymph nodes.*

2.10 Do you use breast cancer multigene signatures for **patients who are diagnosed with local recurrence**? (single answer)

- ☐ Yes
- ☐ No

2.11 Do you use breast cancer multigene signatures in the **neoadjuvant setting**? (single answer)

- ☐ Yes
- ☐ No

2.12 You answered that you use breast cancer multigene signatures in the **neoadjuvant setting. Which, if any, of the following criteria led to that decision**? (one answer per row)

CRITERIA TO USE A BREAST CANCER MULTIGENE SIGNATURE IN NEOADJUVANT SETTING	Never	In selected patients	Routinely
To select patients for clinical trials	☐	☐	☐
To avoid chemotherapy	☐	☐	☐
To define intrinsic subtype	☐	☐	☐

2.13 Do you include patients with micrometastasis in the N0 population that you test? (single answer)

- ☐ Yes
- ☐ No
- ☐ It depends. Please specify: ______

2.14 Have you ever used breast cancer multigene signatures for **other indications** than the ones that are **recommended by clinical guidelines**? (single answer)

- ☐ Yes. Please specify: ____________
- ☐ No

2.15 During the **COVID-19 pandemic situation**, have you **used breast cancer multigene signatures more frequently**? (single answer)

- ☐ Yes, I used them more frequently
    - o Please, specify in which situations: ________________
- ☐ The same than before the COVID-19 pandemic
- ☐ No, I used them less frequently

3 Opinion on the utility of the characteristics of multigene signatures in early breast cancer according to patient profiles

3.1 Regardless of the cost, do you believe that is it useful to know the **tumour subtype** (intrinsic molecular subtypes by gene expression profiling or surrogate intrinsic subtypes by IHC) for **prognosis and treatment decision-making**? Please indicate your level of agreement with the previous quoted statement. 1 being "Useless" and 9 "Essential" (one anwer per row)

INFORMATION	I don't konw	Useless							Must have (Essential)
		1	2	3	4	5	6	7	8	9
Intrinsic molecular subtype by gene expression profiling	☐	☐	☐	☐	☐	☐	☐	☐	☐	☐
Surrogate intrinsic subtype by IHC	☐	☐	☐	☐	☐	☐	☐	☐	☐	☐

3.2 What is the clinical utility of the information provided by the **breast cancer intrinsic molecular subtypes**? 1 being "Useless" and 9 "Essential" (one anwer per row)

INFORMATION PROVIDED	I don't know	Useless								Must have (Essential)
		1	2	3	4	5	6	7	8	9
Assessing prognosis (or residual risk of recurrence with standard of care) of hormone receptor-positive eBC	☐	☐	☐	☐	☐	☐	☐	☐	☐	☐
Avoiding extended administration of endocrine therapy after 5 years	☐	☐	☐	☐	☐	☐	☐	☐	☐	☐
Identifying a group of patients that can safely avoid chemotherapy	☐	☐	☐	☐	☐	☐	☐	☐	☐	☐
Selecting the most appropriate type of chemotherapy treatment	☐	☐	☐	☐	☐	☐	☐	☐	☐	☐
Recruiting a target population in clinical trials	☐	☐	☐	☐	☐	☐	☐	☐	☐	☐

3.2 When you are considering performing a breast cancer multigene signature to **assess the risk of recurrence in eBC**, what degree of importance do you assign to the following characteristics? 1 being "not important at all" and 9 "extremely important (one answer per row)

CHARACTERISTICS OF BREAST CANCER MULTIGENE SIGNATURES	I don't know	Not important at all							Extremely important
		1	2	3	4	5	6	7	8	9
The test has evidence from prospective randomized clinical trials	☐	☐	☐	☐	☐	☐	☐	☐	☐	☐
The test analyses a high number of genes	☐	☐	☐	☐	☐	☐	☐	☐	☐	☐
The test analyses different groups of genes (proliferation, apoptosis, invasion, cell- cycle, hormone-receptor related, etc.)	☐	☐	☐	☐	☐	☐	☐	☐	☐	☐
The test uses FFPE* tissue sample	☐	☐	☐	☐	☐	☐	☐	☐	☐	☐
The test could be performed in the hospital local laboratory	☐	☐	☐	☐	☐	☐	☐	☐	☐	☐
The test provides accurate genomic scores, which allows risk recurrence assessment and chemotherapy-decision	☐	☐	☐	☐	☐	☐	☐	☐	☐	☐
The test takes into consideration clinical parameters along with gene expression	☐	☐	☐	☐	☐	☐	☐	☐	☐	☐
The test relies on retrospective analysis of randomized clinical trials for distant recurrence prognostication	☐	☐	☐	☐	☐	☐	☐	☐	☐	☐
The test relies on retrospective analysis of randomized clinical trials for prediction information	☐	☐	☐	☐	☐	☐	☐	☐	☐	☐
The test provides intrinsic subtypes classification	☐	☐	☐	☐	☐	☐	☐	☐	☐	☐
The test provides quick results availability (less than 1 week)	☐	☐	☐	☐	☐	☐	☐	☐	☐	☐
The test has cost-effectiveness evidence (accurate patient classification that allows the reduction of chemotherapy associated costs	☐	☐	☐	☐	☐	☐	☐	☐	☐	☐
The test has regulatory endorsement from the FDA or local authorities	☐	☐	☐	☐	☐	☐	☐	☐	☐	☐
The test has regulatory endorsement from CE-IVD	☐	☐	☐	☐	☐	☐	☐	☐	☐	☐

The test is included in and recommended by national or international guidelines	☐	☐	☐	☐	☐	☐	☐	☐	☐	☐

**CE-IVD: CE-Marking for In-Vitro Diagnostic Devices Directive; FDA: US Food and Drug Administration; FFPE: Formalin-fixed paraffin-embedded*

3.4 How important are the **prognostic value** from a breast cancer multigene signature when deciding **therapeutic choices** to avoid distant recurrence (within 10 years) and late distant recurrence (within 5-10 years) in patients with eBC? 1 being "Not important at all" and 9 "Extremely important" (one answer per row)

PATIENT PROFILE	Type of therapy	I don't know	Not important at all							Extremely important
			1	2	3	4	5	6	7	8	9
In eBC patients with node-negative disease	*Chemotherapy in the adjuvant setting*	☐	☐	☐	☐	☐	☐	☐	☐	☐	☐
	Extended endocrine therapy	☐	☐	☐	☐	☐	☐	☐	☐	☐	☐
In eBC patients with 1 to 3 positive nodes	*Chemotherapy in the adjuvant setting*	☐	☐	☐	☐	☐	☐	☐	☐	☐	☐
	Extended endocrine therapy	☐	☐	☐	☐	☐	☐	☐	☐	☐	☐

**eBC: early breast cancer ER+/HER2-*

3.5 What **utility** do you allocate to breast cancer multigene signatures in the **following settings**? 1 being "Useless" and 9 "Essential" (one answer per row)

NOTE: We acknowledge that the information provided by each individual multigene signature may be different, but if you think that at least one or more tests provide information on each one of the topics listed below, please answer the question thinking about such test/s.

Situation	I don't know	Useless							Essential
		1	2	3	4	5	6	7	8	9
Patients with pre-menopausal eBC	☐	☐	☐	☐	☐	☐	☐	☐	☐	☐
Patients with post-menopausal eBC	☐	☐	☐	☐	☐	☐	☐	☐	☐	☐
Male patients with eBC	☐	☐	☐	☐	☐	☐	☐	☐	☐	☐
Patients with eBC and a HER2 overexpressed profile	☐	☐	☐	☐	☐	☐	☐	☐	☐	☐
Patients with eBC and triple-negative disease	☐	☐	☐	☐	☐	☐	☐	☐	☐	☐
Other histological eBC subtypes beyond invasive ductal carcinoma	☐	☐	☐	☐	☐	☐	☐	☐	☐	☐
In patients with eBC before neoadjuvant treatment	☐	☐	☐	☐	☐	☐	☐	☐	☐	☐

In the neoadjuvant setting	☐	☐	☐	☐	☐	☐	☐	☐	☐	☐
In the metastatic setting	☐	☐	☐	☐	☐	☐	☐	☐	☐	☐

**eBC: early breast cancer.*

4 Recommendations on the use of breast cancer multigene signatures in clinical practice

4.1 Please indicate your level of agreement with the following statements related to breast cancer multigene signatures recommendations. 1 being "Fully disagree" and 9 "Fully agree" (1 answer per row)

STATEMENTS	I don't know	Fully disagree							Fully agree
		1	2	3	4	5	6	7	8	9
In addition to genomic results, we must also take into consideration clinical and pathological features of the disease	☐	☐	☐	☐	☐	☐	☐	☐	☐	☐
The breast cancer multigene signature must provide information based on evidence from prospective randomized clinical trials	☐	☐	☐	☐	☐	☐	☐	☐	☐	☐
The breast cancer multigene signature must provide both prognostic and predictive information	☐	☐	☐	☐	☐	☐	☐	☐	☐	☐
The breast cancer multigene signature must provide accurate results to guide adjuvant chemotherapy decision on a cost-effective way	☐	☐	☐	☐	☐	☐	☐	☐	☐	☐
The breast cancer multigene signature must provide information on the risk of late distant recurrence from 5 to 10 years	☐	☐	☐	☐	☐	☐	☐	☐	☐	☐
The breast cancer multigene signature must provide the intrinsic subtype information	☐	☐	☐	☐	☐	☐	☐	☐	☐	☐
Second generation of breast cancer multigene signatures provide more accurate assessment of the risk of recurrence and prognostic result values	☐	☐	☐	☐	☐	☐	☐	☐	☐	☐
The results from the breast cancer multigene signature must be available quickly (less than 1 week)	☐	☐	☐	☐	☐	☐	☐	☐	☐	☐
Second generation of breast cancer multigene signatures enhance the prognostic value	☐	☐	☐	☐	☐	☐	☐	☐	☐	☐

for distant recurrence and risk stratification in node-positive disease									

4.2 Please indicate your level of agreement with the following **recommendations in relation to** the **patient profiles that could benefit from breast cancer multigene signatures**. 1 being "Fully disagree" and 9 "Fully agree" (1 answer per row)

RECOMMENDATIONS	I don't know	Fully disagree							Fully agree
		1	2	3	4	5	6	7	9
Breast cancer multigene signatures must be performed in all patients when breast cancer is suspected	☐	☐	☐	☐	☐	☐	☐	☐	☐
Breast cancer multigene signatures must be performed in all patients when early breast cancer is diagnosed/suspected to plan locoregional or systemic treatment approach	☐	☐	☐	☐	☐	☐	☐	☐	☐
Breast cancer Multigene multigene signatures must be performed in all ER+/HER2- early breast cancer patients after surgery to define both the risk of recurrence and the most suitable treatment	☐	☐	☐	☐	☐	☐	☐	☐	☐
Breast cancer multigene signatures must be repeated in all ER+/HER2- early breast cancer patients when a locoregional recurrence occurs	☐	☐	☐	☐	☐	☐	☐	☐	☐

4.3 Please indicate your level of agreement with the following **general recommendations**. 1 being "Fully disagree" and 9 "Fully agree" (1 answer per row)

RECOMMENDATIONS	I don't know	Fully disagree								Fully agree
		1	2	3	4	5	6	7	8	9
Hospitals must define a policy for the use of breast cancer multigene signatures according to national and or international guidelines	☐	☐	☐	☐	☐	☐	☐	☐	☐	☐
There is a need to train oncologists on breast cancer multigene signatures	☐	☐	☐	☐	☐	☐	☐	☐	☐	☐
There is a need to train pathologists on breast cancer multigene signatures	☐	☐	☐	☐	☐	☐	☐	☐	☐	☐
There is a need to train nurse specialists on breast cancer multigene signatures	☐	☐	☐	☐	☐	☐	☐	☐	☐	☐
There is a need to better educate patients on breast cancer multigene signatures utility	☐	☐	☐	☐	☐	☐	☐	☐	☐	☐

Patients have the right to access to the results of breast cancer multigene signatures in order to participate in the treatment decision making process	☐	☐	☐	☐	☐	☐	☐	☐	☐	☐

4.4 Please indicate your level of agreement with the following statements on the **discordance between IHC-based surrogate subtypes and PAM50 intrinsic molecular subtypes**. 1 being "Fully disagree" and 9 "Fully agree" (1 answer per row)

STATEMENTS	I don't know	Fully disagree							Fully agree
		1	2	3	4	5	6	7	8	9
Current immunohistochemical (IHC)-based definitions of luminal A and B breast cancers are imperfect when compared with multigene expr ession-based assays	☐	☐	☐	☐	☐	☐	☐	☐	☐	☐
The intrinsic subtype classification based on IHC are not sufficient to show an adequate surrogate for the genomic subtypes	☐	☐	☐	☐	☐	☐	☐	☐	☐	☐
The discordance between IHC-based subtype and PAM50 intrinsic subtypes, could explain the under-treatment or over-treatment of breast cancer patients	☐	☐	☐	☐	☐	☐	☐	☐	☐	☐
The intrinsic subtype classification provided by PAM50 supports its clinical utility	☐	☐	☐	☐	☐	☐	☐	☐	☐	☐

**IHC: immunohistochemistry.*

5 Future applications of breast cancer multigene signatures

5.1 Please indicate your level of agreement with the **need for validated breast cancer multigene signatures for risk of distant recurrence and prediction of treatment benefit in the following settings**. 1 being "Fully disagree" and 9 "Fully agree" (1 answer per row)

There is a need for validated breast cancer multigene signatures in the following settings:	Assessment provided	I don't know	Fully disagree								Fully agree
			1	2	3	4	5	6	7	8	9
ER+ advanced and/or metastatic breast cancer	*Prognosis*	☐	☐	☐	☐	☐	☐	☐	☐	☐	☐
	Predict treatment benefit	☐	☐	☐	☐	☐	☐	☐	☐	☐	☐
HER2+ early breast cancer	*Risk of recurrence*	☐	☐	☐	☐	☐	☐	☐	☐	☐	☐
	Predict treatment benefit	☐	☐	☐	☐	☐	☐	☐	☐	☐	☐
HER2+ advanced breast cancer	*Prognosis*	☐	☐	☐	☐	☐	☐	☐	☐	☐	☐

	Predict treatment benefit	☐	☐	☐	☐	☐	☐	☐	☐	☐	☐
Triple Negative early breast cancer	*Risk of recurrence*	☐	☐	☐	☐	☐	☐	☐	☐	☐	☐
	Predict treatment benefit	☐	☐	☐	☐	☐	☐	☐	☐	☐	☐
Triple Negative advanced breast cancer	*Prognosis*	☐	☐	☐	☐	☐	☐	☐	☐	☐	☐
	Predict treatment benefit	☐	☐	☐	☐	☐	☐	☐	☐	☐	☐
Neoadjuvant	*Risk of recurrence*	☐	☐	☐	☐	☐	☐	☐	☐	☐	☐
	Predict treatment benefit	☐	☐	☐	☐	☐	☐	☐	☐	☐	☐

**ER: oestrogen receptor.*

PROCURE questionnaire – Wave 2

Thank you very much for your willingness to participate in the **PROCURE Project**.

Of the **5 initial sections** into which the questionnaire was divided, the first two will not be asked again (excepting 3 questions from Section 2). In the remaining three sections only those questions where consensus was not reached in Wave 1 will be asked again. Consensus has already been reached in **37%** of the items asked during Wave 1, **therefore Wave 2 will be much shorter than the previous questionnaire. It won't take you more than 30 minutes to answer all questions.**

1. Participant's profile (this section will not be asked again in the 2nd wave).
2. Current daily clinical practice with breast cancer multigene signatures (this section will not be asked again in the 2nd wave).
    a. Three questions of this section Q2.2; Q2.3 and Q2.7 will be asked again.
3. Opinions on the utility of the characteristics of multigene signatures in early breast cancer according to patient profiles.
4. Recommendations on the use of breast cancer multigene signatures in clinical practice.
5. Future applications of breast cancer multigene signatures.

In addition, the results from the Wave 1 will be displayed for each of the questions. As a reminder, **consensus was initially defined as reached when 70% or more of the participants agree or disagree on the same statement.**

We would like to remind you that your answers will always be kept confidential, thus we would appreciate it if you could answer as honestly as possible. Please epxress your level of agreement or disagreement with the different statements based on your experience and professional opinion.

Finally, we want to inform you that **Wave 2 questionnaire will be open until May 30th, 2021.** The platform allows to save your progress, so you will be able to access the questionnaire as many times as necessary to complete it during this period. Once the end of the period reached, access to the questionnaire will be closed.

Once again, thank you for your commitment and time! Let's start!

2 Current daily clinical practice with breast cancer multigene signatures*

Taking into consideration newly available data recently pusblished, we kindly ask to answer again these 3 questions regarding your current clinical practice.

2.2 Is there a **hospital/country guideline** in your hospital/region that defines when to use a breast cancer multigene signature? (single answer)

- ☐ Yes
- ☐ No

2.3 Taking into consideration the **new results recently published**, what are the **criteria defined for the use of breast cancer multigene signatures** in your hospital? (multiple answer)

- ☐ Age of the patient
- ☐ Menopausal status
- ☐ Tumour size
- ☐ Nodal status
- ☐ Spectrum of % of ER expression
- ☐ Spectrum of % of PR expression
- ☐ HER2 negative by IHC/FISH/CISH
- ☐ Clinical-pathological algorithms (Adjuvant Online, NPI, Predict, etc.)
- ☐ Grade of the tumour
- ☐ % of Ki67 expression
- ☐ Vascular infiltration of the tumour
- ☐ Uncertainty about benefit of chemotherapy
- ☐ Luminal B surrogate breast cancer
- ☐ Uncertainty about endocrine therapy benefit
- ☐ Other. Please, specify: ____________

**CISH: chromogenic in-situ hybridisation; ER: Oestrogen receptor; FISH: fluorescent in-situ hybridization; IHC: inmunohistochemistry; PR: progesterone receptor.*

2.7 **At the light of the new data recently published**, when do you **use a breast cancer multigene signature to define the prognosis and treatment needs** in the following ER+/HER2- early breast cancer profiles? (one answer per row)

PATIENT PROFILES	Never	In selected patients	Routinely
Gender			
Male	☐	☐	☐
Female	☐	☐	☐
Age			
< 40 years old	☐	☐	☐
40 – 50 years old	☐	☐	☐
> 50 years old	☐	☐	☐
Menopausal status			
Pre-menopausal status	☐	☐	☐
Post-menopausal status	☐	☐	☐
Nodal status			
Negative LN	☐	☐	☐
1 to 3 positive LN	☐	☐	☐
≥ 4 positive LN	☐	☐	☐
HR status			
HR positive	☐	☐	☐
HR negative	☐	☐	☐
HER2 status			

HER2 positive	☐	☐	☐
HER2 negative	☐	☐	☐
Triple negative	☐	☐	☐

**HR: hormone receptor; LN: lymph nodes.*

3 Opinion on the utility of the characteristics of multigene signatures in early breast cancer according to patient profiles

Here we show you the aggregated results obtained after analysing Wave 1 answers regarding the opinion of participants on the utility of the characteristics of multigene signatures in early breast cancer according to patient profiles. In addition, the highest % of agreement or disagreement reached in the Wave 1 is also displayed.

In Wave 2, we ask you to answer again, only those items/phrases where a consensus has not been reached in Wave 1. These items have been highlighted in grey.

Please answer all questions as honestly as possible.

Thank you,

3.1 Regardless of the cost, do you believe that is it useful to know the **tumour subtype** (intrinsic molecular subtypes by gene expression profiling or surrogate intrinsic subtypes by IHC) for **prognosis and treatment decision-making**? Please indicate your level of agreement with the previous quoted statement. 1 being “Useless” and 9 “Essential” (one anwer per row)

INFORMATION	I don’t konw	Useless							Must have (Essential)	Wave 1 Results
		1	2	3	4	5	6	7	8	9	
Intrinsic molecular subtype by gene expression profiling	☐	☐	☐	☐	☐	☐	☐	☐	☐	☐	**66%** agreement (7-9)
Surrogate intrinsic subtype by IHC	☐	☐	☐	☐	☐	☐	☐	☐	☐	☐	**59%** agreement (7-9)

3.2 What is the clinical utility of the information provided by the **breast cancer intrinsic molecular subtypes**? 1 being “Useless” and 9 “Essential” (one anwer per row)

INFORMATION PROVIDED	I don’t know	Useless								Must have (Essential)	Wave 1 Results
		1	2	3	4	5	6	7	8	9	
Assessing prognosis (or residual risk of recurrence with standard of care) of hormone receptor-positive eBC	☐	☐	☐	☐	☐	☐	☐	☐	☐	☐	**75%** agreement (7-9)
Avoiding extended administration of endocrine therapy after 5 years	☐	☐	☐	☐	☐	☐	☐	☐	☐	☐	**38%** neutral (4-6)
Identifying a group of patients that can safely avoid chemotherapy	☐	☐	☐	☐	☐	☐	☐	☐	☐	☐	**77%** agreement (7-9)
Selecting the most appropriate type of chemotherapy treatment	☐	☐	☐	☐	☐	☐	☐	☐	☐	☐	**46%** disagreement (1-3)

Recruiting a target population in clinical trials	☐	☐	☐	☐	☐	☐	☐	☐	☐	☐	**46%** agreement (7-9)

3.2 When you are considering performing a breast cancer multigene signature to **assess the risk of recurrence in eBC**, what degree of importance do you assign to the following characteristics? 1 being "not important at all" and 9 "extremely important (one answer per row)

CHARACTERISTICS OF BREAST CANCER MULTIGENE SIGNATURES	I don't know	Not important at all							Extremely important	Wave 1 Results
		1	2	3	4	5	6	7	8	9	
The test has evidence from prospective randomized clinical trials	☐	☐	☐	☐	☐	☐	☐	☐	☐	☐	**88%** agreement (7-9)
The test analyses a high number of genes	☐	☐	☐	☐	☐	☐	☐	☐	☐	☐	**51%** neutral (4-6)
The test analyses different groups of genes (proliferation, apoptosis, invasion, cell- cycle, hormone-receptor related, etc.)	☐	☐	☐	☐	☐	☐	☐	☐	☐	☐	**48%** agreement (7-9)
The test uses FFPE* tissue sample	☐	☐	☐	☐	☐	☐	☐	☐	☐	☐	**72%** agreement (7-9)
The test could be performed in the hospital local laboratory	☐	☐	☐	☐	☐	☐	☐	☐	☐	☐	**57%** agreement (7-9)
The test provides accurate genomic scores, which allows risk recurrence assessment and chemotherapy-decision	☐	☐	☐	☐	☐	☐	☐	☐	☐	☐	**84%** agreement (7-9)
The test takes into consideration clinical parameters along with gene expression	☐	☐	☐	☐	☐	☐	☐	☐	☐	☐	**72%** agreement (7-9)
The test relies on retrospective analysis of randomized clinical trials for distant recurrence prognostication	☐	☐	☐	☐	☐	☐	☐	☐	☐	☐	**43%** agreement (7-9)
The test relies on retrospective analysis of randomized clinical trials for prediction information	☐	☐	☐	☐	☐	☐	☐	☐	☐	☐	**43%** agreement (7-9)
The test provides intrinsic subtypes classification	☐	☐	☐	☐	☐	☐	☐	☐	☐	☐	**55%** agreement (7-9)

The test provides quick results availability (less than 1 week)	☐	☐	☐	☐	☐	☐	☐	☐	☐	☐	**72%** agreement (7-9)
The test has cost-effectiveness evidence (accurate patient classification that allows the reduction of chemotherapy associated costs	☐	☐	☐	☐	☐	☐	☐	☐	☐	☐	**70%** Agreement (7-9)
The test has regulatory endorsement from the FDA or local authorities	☐	☐	☐	☐	☐	☐	☐	☐	☐	☐	**71%** agreement (7-9)
The test has regulatory endorsement from CE-IVD	☐	☐	☐	☐	☐	☐	☐	☐	☐	☐	**70%** agreement (7-9)
The test is included in and recommended by national or international guidelines	☐	☐	☐	☐	☐	☐	☐	☐	☐	☐	**85%** Agreement (7-9)

**CE-IVD: CE-Marking for In-Vitro Diagnostic Devices Directive; FDA: US Food and Drug Administration; FFPE: Formalin-fixed paraffin-embedded*

3.4 How important are the **prognostic value** from a breast cancer multigene signature when deciding **therapeutic choices** to avoid distant recurrence (within 10 years) and late distant recurrence (within 5-10 years) in patients with eBC? 1 being "Not important at all" and 9 "Extremely important" (one answer per row)

PATIENT PROFILE	Type of therapy	I don't know	Not important at all								Extremely important	Wave 1 Results
			1	2	3	4	5	6	7	8	9	
In eBC patients with node-negative disease	*Chemotherapy in the adjuvant setting*	☐	☐	☐	☐	☐	☐	☐	☐	☐	☐	**89%** agreement (7-9)
	Extended endocrine therapy	☐	☐	☐	☐	☐	☐	☐	☐	☐	☐	**53%** agreement (7-9)
In eBC patients with 1 to 3 positive nodes	*Chemotherapy in the adjuvant setting*	☐	☐	☐	☐	☐	☐	☐	☐	☐	☐	**75%** agreement (7-9)
	Extended endocrine therapy	☐	☐	☐	☐	☐	☐	☐	☐	☐	☐	**48%** agreement (7-9)

**eBC: early breast cancer ER+/HER2-*

3.5 What **utility** do you allocate to breast cancer multigene signatures in the **following settings**? 1 being "Useless" and 9 "Essential" (one answer per row)

NOTE: We acknowledge that the information provided by each individual multigene signature may be different, but if you think that at least one or more tests provide information on each one of the topics listed below, please answer the question thinking about such test/s.

Situation	I don't know	Useless							Essential	Wave 1 Results
		1	2	3	4	5	6	7	8	9	
Patients with pre-menopausal eBC	☐	☐	☐	☐	☐	☐	☐	☐	☐	☐	**60%** agreement (7-9)
Patients with post-menopausal eBC	☐	☐	☐	☐	☐	☐	☐	☐	☐	☐	**90%** agreement (7-9)
Male patients with eBC	☐	☐	☐	☐	☐	☐	☐	☐	☐	☐	**34%** agreement (7-9)
Patients with eBC and a HER2 overexpressed profile	☐	☐	☐	☐	☐	☐	☐	☐	☐	☐	**69%** disagreement (1-3)
Patients with eBC and triple-negative disease	☐	☐	☐	☐	☐	☐	☐	☐	☐	☐	**73%** disagreement (1-3)
Other histological eBC subtypes beyond invasive ductal carcinoma	☐	☐	☐	☐	☐	☐	☐	☐	☐	☐	**39%** agreement (7-9)
In patients with eBC before neoadjuvant treatment	☐	☐	☐	☐	☐	☐	☐	☐	☐	☐	**36%** neutral (4-6)
In the neoadjuvant setting	☐	☐	☐	☐	☐	☐	☐	☐	☐	☐	**39%** disagreement (1-3)
In the metastatic setting	☐	☐	☐	☐	☐	☐	☐	☐	☐	☐	**53%** disagreement (1-3)

**eBC: early breast cancer.*

4 Recommendations on the use of breast cancer multigene signatures in clinical practice

Here we show you the results obtained after analysing Wave 1 answers regarding the opinion of particpants regarding recommendations on the use of breast cancer multigene signatures in clinical practice. In addition the highest % of agreement or disagreement reached in the Wave 1 is also displayed.

In Wave 2, we ask you to answer again, only those items/phrases where a consensus has not been reached in Wave 1. These items have been highlighted in grey.

Please answer all questions as honestly as possible.

Thank you,

4.1 Please indicate your level of agreement with the following statements related to **breast cancer multigene signatures recommendations**. 1 being "Fully disagree" and 9 "Fully agree" (1 answer per row)

STATEMENTS	I don't know	Fully disagree							Fully agree	Wave 1 Results
		1	2	3	4	5	6	7	8	9	
In addition to genomic results, we must also take into consideration clinical and pathological features of the disease	☐	☐	☐	☐	☐	☐	☐	☐	☐	☐	**90%** agreement (7-9)
The breast cancer multigene signature must provide	☐	☐	☐	☐	☐	☐	☐	☐	☐	☐	**85%**

information based on evidence from prospective randomized clinical trials											agreement (7-9)
The breast cancer multigene signature must provide both prognostic and predictive information	☐	☐	☐	☐	☐	☐	☐	☐	☐	☐	**81%** agreement (7-9)
The breast cancer multigene signature must provide accurate results to guide adjuvant chemotherapy decision on a cost-effective way	☐	☐	☐	☐	☐	☐	☐	☐	☐	☐	**81%** agreement (7-9)
The breast cancer multigene signature must provide information on the risk of late distant recurrence from 5 to 10 years	☐	☐	☐	☐	☐	☐	☐	☐	☐	☐	**67%** agreement (7-9)
The breast cancer multigene signature must provide the intrinsic subtype information	☐	☐	☐	☐	☐	☐	☐	☐	☐	☐	**52%** agreement (7-9)
Second generation of breast cancer multigene signatures provide more accurate assessment of the risk of recurrence and prognostic result values	☐	☐	☐	☐	☐	☐	☐	☐	☐	☐	**59%** agreement (7-9)
The results from the breast cancer multigene signature must be available quickly (less than 1 week)	☐	☐	☐	☐	☐	☐	☐	☐	☐	☐	**63%** agreement (7-9)
Second generation of breast cancer multigene signatures enhance the prognostic value for distant recurrence and risk stratification in node-positive disease	☐	☐	☐	☐	☐	☐	☐	☐	☐	☐	**60%** agreement (7-9)

4.2 Please indicate your level of agreement with the following **recommendations in relation to** the **patient profiles that could benefit from breast cancer multigene signatures**. 1 being "Fully disagree" and 9 "Fully agree" (1 answer per row)

RECOMMENDATIONS	I don't know	Fully disagree 1	2	3	4	5	6	7	8	Fully agree 9	Wave 1 Results
Breast cancer multigene signatures must be performed in all patients when breast cancer is suspected	☐	☐	☐	☐	☐	☐	☐	☐	☐	☐	**77%** disagreement (1-3)
Breast cancer multigene signatures must be performed in all patients when early breast cancer is diagnosed/suspected to plan locoregional or systemic treatment approach	☐	☐	☐	☐	☐	☐	☐	☐	☐	☐	**69%** agreement (7-9)
Breast cancer Multigene multigene signatures must be	☐	☐	☐	☐	☐	☐	☐	☐	☐	☐	**45%**

performed in all ER+/HER2- early breast cancer patients after surgery to define both the risk of recurrence and the most suitable treatment											disagreement (1-3)
Breast cancer multigene signatures must be repeated in all ER+/HER2- early breast cancer patients when a locoregional recurrence occurs	☐	☐	☐	☐	☐	☐	☐	☐	☐	☐	**62%** disagreement (1-3)

4.3 Please indicate your level of agreement with the following **general recommendations**. 1 being "Fully disagree" and 9 "Fully agree" (1 answer per row)

RECOMMENDATIONS	I don't know	Fully disagree								Fully agree	Wave 1 Results
		1	2	3	4	5	6	7	8	9	
Hospitals must define a policy for the use of breast cancer multigene signatures according to national and or international guidelines	☐	☐	☐	☐	☐	☐	☐	☐	☐	☐	**77%** agreement (7-9)
There is a need to train oncologists on breast cancer multigene signatures	☐	☐	☐	☐	☐	☐	☐	☐	☐	☐	**84%** agreement (7-9)
There is a need to train pathologists on breast cancer multigene signatures	☐	☐	☐	☐	☐	☐	☐	☐	☐	☐	**82%** agreement (7-9)
There is a need to train nurse specialists on breast cancer multigene signatures	☐	☐	☐	☐	☐	☐	☐	☐	☐	☐	**50%** agreement (7-9)
There is a need to better educate patients on breast cancer multigene signatures utility	☐	☐	☐	☐	☐	☐	☐	☐	☐	☐	**62%** agreement (7-9)
Patients have the right to access to the results of breast cancer multigene signatures in order to participate in the treatment decision making process	☐	☐	☐	☐	☐	☐	☐	☐	☐	☐	**85%** agreement (7-9)

4.4 Please indicate your level of agreement with the following statements on the **discordance between IHC-based surrogate subtypes and PAM50 intrinsic molecular subtypes**. 1 being "Fully disagree" and 9 "Fully agree" (1 answer per row)

STATEMENTS	I don't know	Fully disagree								Fully agree	Wave 1 Results
		1	2	3	4	5	6	7	8	9	
Current immunohistochemical (IHC)-based definitions of luminal A and B breast cancers are imperfect when compared with multigene expr ession-based assays	☐	☐	☐	☐	☐	☐	☐	☐	☐	☐	**72%** agreement (7-9)
The intrinsic subtype classification based on IHC are not sufficient to	☐	☐	☐	☐	☐	☐	☐	☐	☐	☐	**65%** agreement (7-9)

show an adequate surrogate for the genomic subtypes										
The discordance between IHC-based subtype and PAM50 intrinsic subtypes, could explain the under-treatment or over-treatment of breast cancer patients	☐	☐	☐	☐	☐	☐	☐	☐	☐	**65%** agreement (7-9)
The intrinsic subtype classification provided by PAM50 supports its clinical utility	☐	☐	☐	☐	☐	☐	☐	☐	☐	**70%** agreement (7-9)

**IHC: immunohistochemistry.*

5 Future applications of breast cancer multigene signatures

Here we show you the results obtained after analysing Wave 1 answers regarding the opinion of particpants on future application of breast cancer multigene signatures. In addition, the highest % of agreement or disagreement reached in the Wave 1 is also displayed.

In Wave 2, we ask you to answer again, only those items/phrases where a consensus has not been reached in Wave 1. These items have been highlighted in grey.

Please answer all questions as honestly as possible.

Thank you,

5.1 **Thinking about the need of developing new breast cancer multigene signatures**, please indicate your level of agreement with the **need for validated BCMS to evaluate the risk of distant recurrence and predict treatment benefit in the following settings**. 1 being "Fully disagree" and 9 "Fully agree" (1 answer per row)

There is a need for validated breast cancer multigene signatures in the following settings:	Assessment provided	I don't know	Fully disagree							Fully agree	Wave 1 Results
			1	2	3	4	5	6	7	8	9	
ER+ advanced and/or metastatic breast cancer	*Prognosis*	☐	☐	☐	☐	☐	☐	☐	☐	☐	☐	**41%** agreement (7-9)
	Predict treatment benefit	☐	☐	☐	☐	☐	☐	☐	☐	☐	☐	**63%** agreement (7-9)
HER2+ early breast cancer	*Risk of recurrence*	☐	☐	☐	☐	☐	☐	☐	☐	☐	☐	**50%** agreement (7-9)
	Predict treatment benefit	☐	☐	☐	☐	☐	☐	☐	☐	☐	☐	**55%** agreement (7-9)
HER2+ advanced breast cancer	*Prognosis*	☐	☐	☐	☐	☐	☐	☐	☐	☐	☐	**32%** neutral (4-6)
	Predict treatment benefit	☐	☐	☐	☐	☐	☐	☐	☐	☐	☐	**43%** agreement (7-9)
	Risk of recurrence	☐	☐	☐	☐	☐	☐	☐	☐	☐	☐	**47%** agreement (7-9)

Triple Negative early breast cancer	*Predict treatment benefit*	☐	☐	☐	☐	☐	☐	☐	☐	☐	☐	**50%** agreement (7-9)
Triple Negative advanced breast cancer	*Prognosis*	☐	☐	☐	☐	☐	☐	☐	☐	☐	☐	**33%** agreement (7-9)
	Predict treatment benefit	☐	☐	☐	☐	☐	☐	☐	☐	☐	☐	**48%** agreement (7-9)
Neoadjuvant	*Risk of recurrence*	☐	☐	☐	☐	☐	☐	☐	☐	☐	☐	**60%** agreement (7-9)
	Predict treatment benefit	☐	☐	☐	☐	☐	☐	☐	☐	☐	☐	**67%** agreement (7-9)

**ER: oestrogen receptor.*

Supplementary References

1. ASCO: Andre F, Ismaila N, Allison KH, Barlow WE, Collyar DE, Damodaran S, Henry NL, Jhaveri K, Kalinsky K, Kuderer NM, Litvak A, Mayer EL, Pusztai L, Raab R, Wolff AC, Stearns V. Biomarkers for Adjuvant Endocrine and Chemotherapy in Early-Stage Breast Cancer: ASCO Guideline Update. J Clin Oncol. 2022 Jun 1;40(16):1816-1837. doi: 10.1200/JCO.22.00069. Epub 2022 Apr 19. Erratum in: J Clin Oncol. 2022 Aug 1;40(22):2514. PMID: 35439025.

2. NCCN: National Comprehensive Cancer Network. NCCN Guidelines: Breast cancer v4.2022. (2022).

3. ESMO: Cardoso, F. et al. Early breast cancer: ESMO Clinical Practice Guidelines for diagnosis, treatment, and follow-up. Annals of Oncology 30, 1194–1220 (2019).
