# Supplementary material for: PROCURE European consensus on breast cancer multigene signatures in early breast cancer management
Source: NPJ Breast Cancer. 2023 Feb 24;9:8. doi: 10.1038/s41523-023-00510-9 (PMC9951144; doi:10.1038/s41523-023-00510-9)
Supplement: Supplementary file 2 — Reporting Summary [file 41523_2023_510_MOESM2_ESM.pdf]

Corresponding author(s): Curigliano, Giuseppe

Last updated by author(s): Jan 10, 2023

Reporting Summary

Nature Portfolio wishes to improve the reproducibility of the work that we publish. This form provides structure for consistency and transparency in reporting. For further information on Nature Portfolio policies, see our Editorial Policies and the Editorial Policy Checklist.

Statistics

For all statistical analyses, confirm that the following items are present in the figure legend, table legend, main text, or Methods section.

n/a	Confirmed	
☒	☐	The exact sample size (*n*) for each experimental group/condition, given as a discrete number and unit of measurement
☒	☐	A statement on whether measurements were taken from distinct samples or whether the same sample was measured repeatedly
☒	☐	The statistical test(s) used AND whether they are one- or two-sided<br>*Only common tests should be described solely by name; describe more complex techniques in the Methods section.*
☒	☐	A description of all covariates tested
☒	☐	A description of any assumptions or corrections, such as tests of normality and adjustment for multiple comparisons
☒	☐	A full description of the statistical parameters including central tendency (e.g. means) or other basic estimates (e.g. regression coefficient) AND variation (e.g. standard deviation) or associated estimates of uncertainty (e.g. confidence intervals)
☒	☐	For null hypothesis testing, the test statistic (e.g. F, t, r) with confidence intervals, effect sizes, degrees of freedom and P value noted<br>*Give P values as exact values whenever suitable.*
☒	☐	For Bayesian analysis, information on the choice of priors and Markov chain Monte Carlo settings
☒	☐	For hierarchical and complex designs, identification of the appropriate level for tests and full reporting of outcomes
☒	☐	Estimates of effect sizes (e.g. Cohen's *d*, Pearson's *r*), indicating how they were calculated

Our web collection on statistics for biologists contains articles on many of the points above.

Software and code

Policy information about availability of computer code

Data collection	No software was used
Data analysis	No software was used

For manuscripts utilizing custom algorithms or software that are central to the research but not yet described in published literature, software must be made available to editors and reviewers. We strongly encourage code deposition in a community repository (e.g. GitHub). See the Nature Portfolio guidelines for submitting code & software for further information.

Data

Policy information about availability of data

All manuscripts must include a data availability statement. This statement should provide the following information, where applicable:

- Accession codes, unique identifiers, or web links for publicly available datasets
- A description of any restrictions on data availability
- For clinical datasets or third party data, please ensure that the statement adheres to our policy

The full data set is available from the corresponding author upon request.

Human research participants

Policy information about studies involving human research participants and Sex and Gender in Research.

Reporting on sex and gender	Gender was determined based on self-reporting and consent has been obtained from participants. The use of this information was used just to characterise the sample, no infererences or gender based analysis had been done because it was not relevant for our study. We just wanted to know the opinion from participants regarding the topic from our Delphi study.
Population characteristics	Age and gender were asked on the Delphi questionnaire for the sole purpose of characterising the sample. No analysis of participants' responses by gender or age was conducted. The criteria for inclusion of participants were defined by the scientific committee of the project. The criteria were as follows: 1) experience in breast cancer (≥5 years); 2) high volume (>50%) of patients with eBC; 3) at least 1 year of experience with BCMS; and 4) practice in large public hospitals. A balanced sample of 163 European clinicians were invited to participate in the Delphi survey.
Recruitment	Participants were invited to participate via email.
Ethics oversight	There was no protocol for this study.

Note that full information on the approval of the study protocol must also be provided in the manuscript.

Field-specific reporting

Please select the one below that is the best fit for your research. If you are not sure, read the appropriate sections before making your selection.

☐ Life sciences ☒ Behavioural & social sciences ☐ Ecological, evolutionary & environmental sciences

For a reference copy of the document with all sections, see nature.com/documents/nr-reporting-summary-flat.pdf

Behavioural & social sciences study design

All studies must disclose on these points even when the disclosure is negative.

Study description	This study used the Delphi methodology. The Delphi method allows the collection of opinions to be systematic and to reach consensus on the recommendations put forward. It is structured in two consecutive waves. During Wave 2, only items on which consensus was not reached in Wave 1 were asked again. Its main advantages are the potential to merge diverse information and perspectives, to explore relevant issues and the strong ability to obtain agreement among participating experts, as well as to provide a statistical group response.
Research sample	The PROCURE study was conducted in 11 European countries that were grouped into 5 strategic regions: Iberia (Spain and Portugal), France, United Kingdom, DACH (Germany, Austria, and Switzerland), Italy, and Nordic countries (Denmark, Norway, and Sweden). A balanced sample of 163 European clinicians were invited to participate in the Delphi survey.
Sampling strategy	The sampling strategy was done by convenience The invitation was done by email. For the proper development of the project, a scientific committee consisting of 8 experts in breast cancer and specialised in medical oncology, pathology, or surgery across 8 European countries (Austria, Denmark, France, Germany, Italy, Portugal, Spain, and the United Kingdom) was set up. Members of the scientific committee were selected based on their expertise and their renown within the scientific community to ensure the validity and credibility of the results. Other aspects considered were the number of publications, participation in international and European conferences, and involvement in the development of clinical practice guidelines on breast cancer. At least 1 expert in BCMS from each of the 5 regions considered in the study was included, ensuring region-specific knowledge on use of BCMS and supporting adequate interpretation of the results. This scientific committee reviewed the literature and developed questions and statements to use in the Delphi survey, which is a systematic and iterative approach to build consensus while maintaining anonymity of responders. The scientific committee was responsible for defining the criteria to select the Delphi panellists: 1) experience in breast cancer (≥5 years); 2) high volume (>50%) of patients with eBC; 3) at least 1 year of experience with BCMS; and 4) practice in large public hospitals. A balanced sample of 163 European clinicians were invited to participate in the Delphi survey.
Data collection	Data collection was computer-based, using the Delphi platform created specifically for this project. The panellists registered on the platform through a private access code and defined their user name and password for their profile. The answers were provided by the panellists themselves without the intervention of third parties and the data were treated in a completely anonymised way.
Timing	The Delphi survey was conducted with two consecutive web-based rounds, the first one from December 2020 through February 2021, the second one from April to May 2021.
Data exclusions	163 experts were invited to participate, of which 133 completed the two waves of the Delphi questionnaire. The data of the 30 panellists who did not complete the two waves of the Delphi were excluded from the analysis.

Non-participation	Participation was voluntary, 30 of the 163 participants decided not to continue with the Delphi study and did not complete the second wave of the Delphi. Their data were not included in the analysis.
Randomization	Patients were not randomised into any particular group (randomisation is not applicable to Delphi methodology). Their responses were analysed together.

Reporting for specific materials, systems and methods

We require information from authors about some types of materials, experimental systems and methods used in many studies. Here, indicate whether each material, system or method listed is relevant to your study. If you are not sure if a list item applies to your research, read the appropriate section before selecting a response.

Materials & experimental systems

n/a	Involved in the study
☒	☐ Antibodies
☒	☐ Eukaryotic cell lines
☒	☐ Palaeontology and archaeology
☒	☐ Animals and other organisms
☒	☐ Clinical data
☒	☐ Dual use research of concern

Methods

n/a	Involved in the study
☒	☐ ChIP-seq
☒	☐ Flow cytometry
☒	☐ MRI-based neuroimaging
